# Exploration and analysis of a generalized one-parameter item response model with flexible link functions

**DOI:** 10.3389/fpsyg.2023.1248454

**Published:** 2023-08-30

**Authors:** Xue Wang, Jiwei Zhang, Jing Lu, Guanghui Cheng, Ningzhong Shi

**Affiliations:** ^1^Key Laboratory of Applied Statistics of Ministry of Education (MOE), School of Mathematics and Statistics, Northeast Normal University, Changchun, China; ^2^Faculty of Education, Northeast Normal University, Changchun, China; ^3^Guangzhou Institute of International Finance, Guangzhou University, Guangzhou, China

**Keywords:** Bayesian model evaluation criteria, item response theory, item characteristic curve, one-parameter generalized logistic models, STAN software

## Abstract

This paper primarily analyzes the one-parameter generalized logistic (1PGlogit) model, which is a generalized model containing other one-parameter item response theory (IRT) models. The essence of the 1PGlogit model is the introduction of a generalized link function that includes the probit, logit, and complementary log-log functions. By transforming different parameters, the 1PGlogit model can flexibly adjust the speed at which the item characteristic curve (ICC) approaches the upper and lower asymptote, breaking the previous constraints in one-parameter IRT models where the ICC curves were either all symmetric or all asymmetric. This allows for a more flexible way to fit data and achieve better fitting performance. We present three simulation studies, specifically designed to validate the accuracy of parameter estimation for a variety of one-parameter IRT models using the Stan program, illustrate the advantages of the 1PGlogit model over other one-parameter IRT models from a model fitting perspective, and demonstrate the effective fit of the 1PGlogit model with the three-parameter logistic (3PL) and four-parameter logistic (4PL) models. Finally, we demonstrate the good fitting performance of the 1PGlogit model through an analysis of real data.

## 1. Introduction

Latent trait models, also known as item response theory (IRT) models, have gained widespread application in educational testing and psychological measurement (Lord and Novick, 1968; van der Linden and Hambleton, 1997; Embretson and Reise, 2000; Baker and Kim, 2004). These models utilize the probability of a response to establish the interaction between an examinee's “ability” and the characteristics of the test items, such as difficulty and guessing. The focus is on analyzing the pattern of responses rather than relying on composite or total score variables and linear regression theory. Specifically, IRT aims to model students' ability by examining their performance at the question level, providing a granular perspective on each student's ability based on the unique insights each question offers.

The Rasch model, also known as the one-parameter logistic IRT model, was innovated by Georg Rasch in 1960 and serves as a strategic tool in psychometrics for evaluating categorical data. This data includes responses to reading exams or survey questions and is analyzed in correlation with the trade-off between the respondent's ability, attitude, or personality trait and the item's difficulty (Rasch, 1960). For instance, this model could be used to determine a student's level of reading comprehension or gauge the intensity of a person's stance on issues like capital punishment from their questionnaire responses. Beyond the realms of psychometrics and educational research, the Rasch model and its derivatives also find applications in diverse fields such as healthcare (Bezruczko, 2005), market research (Wright, 1977; Bechtel, 1985), and agriculture (Moral and Rebollo, 2017).

Within the framework of the Rasch model, the probability of a specific response–such as right or wrong–is modeled in relation to the examinee's ability and the item characteristic. Particularly, the classical Rasch model models the probability of a correct response as a logistic function of the discrepancy between the examinee's ability and the item difficulty. Typically, the model parameters depict the proficiency level of examinees and the complexity level of the items on a continuous latent scale. For instance, in educational assessments, the item parameter illustrates the difficulty level, whereas the person parameter represents the ability or attainment level of the examinee. The higher an individual's ability relative to the item difficulty, the higher the probability of a correct response. In cases where an individual's ability position equals the item difficulty level, the Rasch model inherently predicts a 50% chance of a correct response.

Parallel to the logistic IRT models, the normal ogive IRT models utilize the probit function to delineate the relationship between ability and item response, whereas the logistic IRT model employs the logit function to depict the same relationship. This constitutes a fundamental difference between the normal ogive IRT models and the more frequently utilized logistic IRT models. In fact, the use of the normal ogive model in the testing context has been further developed by a number of researchers. Lawley (1943, 1944) was the first to formally employ the normal ogive model to directly model binary item response data. Tucker (1946) used the term “item curve” to indicate the relationship between item response and ability. The early attempts at modeling binary response data culminated in the work of Lord (1952, 1953, 1980) who, unlike the early researchers, treated ability as a latent trait to be estimated and in doing so, laid the foundation for IRT.

The normal ogive IRT models (Lord, 1980; van der Linden and Hambleton, 1997; Embretson and Reise, 2000; Baker and Kim, 2004), also known as the one parameter normal ogive model, are a mathematical model used in the field of psychometrics to relate the latent ability of an examinee to the probability of a correct response on a test item. This model, as a component of IRT, facilitates the design, analysis, and scoring of tests, questionnaires, and comparable instruments intended for the measurement of abilities, attitudes, or other variables.

As previously noted, the Rasch model and the one-parameter normal ogive IRT model are premised upon symmetric functions to delineate the relationship between ability and item response, which result in a symmetric ICC. However, in certain contexts, these symmetric IRT models may not sufficiently capture the characteristics inherent in the data. These situations necessitate the utilization of asymmetric IRT models. Several asymmetric IRT models currently exist, such as the non-parametric Bayesian model, which constructs the ICC with a Dirichlet process prior (Qin, 1998; Duncan and MacEachern, 2008), and the Bayesian beta-mixture IRT model (BBM-IRT), which models the ICC with a flexible finite mixture of beta distribution (Arenson and Karabatsos, 2018). Karabatsos (2016) used the infinite mixture of normal c.d.f to model ICC, while Luzardo and Rodriguez (2015) constructed the ICC using the kernel regression method. There are also some skewed logistic IRT models, such as the logistic positive exponent (LPE) model and the reflection LPE (RLPE) model (Samejima, 1997, 1999, 2000; Bolfarine and Bazan, 2010; Zhang et al., 2022), which utilize skewed modifications of the logit links. Moreover, the positive trait item response model (PTIRM), which employs the log-logistic, lognormal, and Weibull as link functions to link the latent trait to the response, is used in some literature (Lucke, 2014; Magnus and Liu, 2018). In addition, the one-parameter complementary log-log IRT model also yields an asymmetric ICC (Goldstein, 1980; Shim et al., 2022). Compared to their symmetric counterparts, asymmetric IRT models can encapsulate a wider spectrum of data characteristics, particularly when the speed at which the probability of a positive response changes varies across different intervals of the latent trait. Furthermore, asymmetric IRT models are better suited to accommodate data where the probability of a positive response escalates more rapidly at higher trait levels and increases more sluggishly at lower trait levels. These asymmetric models, therefore, have a distinct advantage in capturing the nuanced dynamics of item responses that do not adhere strictly to symmetric patterns, thereby providing a more accurate representation of the interplay between individual ability and item response. As such, they represent a crucial development within the IRT field, broadening the applicability of these models in psychometric analyses and educational measurement.

This article discusses and analyzes the aforementioned one-parameter IRT models: the Rasch model, the one-parameter normal ogive IRT model, and the one-parameter complementary log-log IRT model. We propose a unified model representation that can encompass all three models through the manipulation of specific parameter values. In the present paper, our emphasis is placed on a class of generalized logistic models, introduced initially by Stukel (1988). This class of link functions is guided by a duo of parameters, precisely (η_1_, η_2_). By modulating the values of (η_1_, η_2_), this class is inclusive of logit, probit, complementary log-log link, along with an assortment of other symmetric and asymmetric links as particular instances. This class of models boasts sufficient versatility to accommodate the fitting of identical or diverse links to distinct items nested within the IRT model framework. An additional appealing characteristic of this class streamlines the execution of Markov chain Monte Carlo (MCMC) sampling from the posterior distribution via the recently formulated software, Stan. This research paper encompasses several key aspects. Firstly, we thoroughly discuss symmetric models such as the logit and probit models, as well as asymmetric models like the complementary log-log and generalized logit models, within the framework of a one-parameter IRT model. Secondly, we employ different links for different items in our analysis. Thirdly, we utilize the Stan platform to implement this flexible range of links for one parameter models and provide the corresponding Stan codes. By leveraging Stan, we are able to calculate deviance information criterion (DIC; Spiegelhalter et al., 2002) based on posterior distribution samples, which can naturally guide the selection of links and IRT model types. Lastly, through the 2015 computer-based PISA (Program for International Student Assessment) sciences data, we empirically demonstrate that employing different generalized logit links for different items markedly improves data fit compared to traditional logistic, normal ogive and complementary log-log models, as determined by DIC criteria.

The remainder of this paper is organized as follows. In Section 2, we review the three one-parameter IRT models and the generalized logit link function, then introduce the main model of our study, namely the one-parameter generalized logistic (1PGlogit) model. In Section 3, we describe the Bayesian parameter estimation method that we use, discuss its software implementation, and elaborate on the Bayesian model assessment criteria we employ to evaluate the model fittings. Section 4 presents three simulation studies aimed at exploring the accuracy of model parameter estimation and assessing the fit of the 1PGlogit model in relation to various other symmetric or asymmetric models. In Section 5, we conduct an empirical study to validate the practical utility of the 1PGlogit model. Finally, in Section 6, we provide a summary of the paper.

## 2. Item response theory models with generalized logistic link functions

### 2.1. Overview of the one-parameter IRT models

The initial model in the field of IRT can be traced back to the 1930s, as proposed by Ferguson (1942), Lawley (1943), Mosier (1940, 1941), and Richardson (1936). It was later improved by Lord and Novick (1968) into what is now commonly referred to as the normal ogive model. Suppose we have *N* students each answering *J* items. Let *X* denote the response variable, and let *x*_*ij*_ be the response of the *i*th student (*i* = 1, ⋯  , *N*) on the *j*th item (*j* = 1, ⋯  , *J*). Here, *x*_*ij*_ = 1 indicates a correct answer, and *x*_*ij*_ = 0 indicates an incorrect one. Within the one-parameter normal ogive (1PNO) model, the probability of a correct response by the *i*th student on the *j*th item can be expressed as follows:


(1) $$\begin{array}{l} P\left(x_{ij}=1|\theta_{i},\beta_{j}\right)=\int_{-\infty }^{\theta_{i}-\beta_{j}}\frac{1}{\sqrt{2\pi }}e^{-\frac{z^{2}}{2}}dz. \end{array}$$

Here, β_*j*_ is the difficulty parameter of the *j*th item and θ_*i*_ is the latent trait of the *i*th student. A larger β_*j*_ implies a more difficult item, and the probability of a correct response increases with the increasing value of θ_*i*_. As we can see, the 1PNO model is essentially a generalized linear model with a probit link.

Although the 1PNO model is quite interpretable and intuitive, its computation is complicated. In response to this, Rasch proposed the Rasch model in 1960, which was essentially a generalized linear model with a logit link. Specifically, the probability of a correct response in the model can be expressed in the following form:


(2) $$\begin{array}{l} P\left(x_{ij}=1|\theta_{i},\beta_{j}\right)=\frac{\exp\left(\theta_{i}-\beta_{j}\right)}{1+\exp\left(\theta_{i}-\beta_{j}\right)}, \end{array}$$

where β_*j*_ and θ_*i*_ maintain the same interpretations as in the 1PNO model. In this form, to describe the probability of a student's response, it is no longer necessary to compute the cumbersome integrals, thereby simplifying the calculation.

Both of the models mentioned above possess a symmetrical item characteristic curve (ICC). However, Shim et al. (2022) proposed a one-parameter complementary log-log model (CLLM) which exhibits an asymmetric ICC. The probability of a correct response in the CLLM model can be expressed as follows:


(3) $$\begin{array}{l} P\left(x_{ij}=1|\theta_{i},\beta_{j}\right)=1-\exp\left\{-\exp\left(\theta_{i}-\beta_{j}\right)\right\}, \end{array}$$

where β_*j*_ and θ_*i*_ retain the same interpretations as in the two models discussed earlier. As demonstrated by Shim et al. (2022), the CLLM possesses the capability to effectively address the guessing behavior exhibited by examinees in the three-parameter logistic (3PL) model and, in certain cases, can yield even better results. This implies that CLLM accounts for the effect of guessing. Essentially, the CLLM is a generalized linear model with a complementary log-log link.

### 2.2. Overview of the family of models based on generalized logit links

Let *y* be a dichotomous random variable. We assume that *y* equals 1 with probability μ(η) and 0 with probability 1 − μ(η), where η is a linear predictor. Stukel (1988) introduced a class of generalized logistic models (Glogits), indexed by two shape parameters **λ** = (λ_1_, λ_2_). Therefore, the Glogits model is controlled by a strictly increasing non-linear function *h*_**λ**_ (η). The specific expression is as follows:


(4) $$\begin{array}{l} \mu (\eta )=\frac{\exp\left\{h_{\lambda }\left(\eta \right)\right\}}{1+\exp\left\{h_{\lambda }\left(\eta \right)\right\}}, \end{array}$$

where the function *h*_**λ**_ (η) is defined as follows:

for η > 0 $\left(\mu \left(\eta \right)>\frac{1}{2}\right)$,


(5) $$\begin{array}{l} h_{\lambda }\left(\eta \right)=\{\begin{array}{cc} -\frac{\log(1-\lambda_{1}\eta )}{\lambda_{1}}, & \lambda_{1}<\text{0},\\ \eta , & \lambda_{1}=\text{0},\\ \frac{\exp\left(\lambda_{1}\eta \right)-1}{\lambda_{1}}, & \lambda_{1}>\text{0}. \end{array} \end{array}$$

for η ≤ 0 $\left(\mu \left(\eta \right)\leq \frac{1}{2}\right)$,


(6) $$\begin{array}{l} h_{\lambda }\left(\eta \right)=\{\begin{array}{cc} \frac{\log(1-\lambda_{2}\left|\eta \right|)}{\lambda_{2}}, & \lambda_{2}<\text{0},\\ \eta , & \lambda_{2}=\text{0},\\ -\frac{\exp\left(\lambda_{2}\left|\eta \right|\right)-1}{\lambda_{2}}, & \lambda_{2}>\text{0}. \end{array} \end{array}$$

As evident from the above equations, the logit link serves as a special case of Glogits when λ_1_ = λ_2_ = 0. Furthermore, Stukel (1988) revealed that Glogits can be simplified to several other link functions under certain conditions. For instance, it reduces to a probit link when λ_1_ = λ_2_ ≈ 0.165, a log-log link when λ_1_ ≈ −0.037 and λ_2_ ≈ 0.62, a complementary log-log link when λ_1_ ≈ 0.62 and λ_2_ ≈ −0.037, and a Laplace link when λ_1_ = λ_2_ ≈ −0.077.

### 2.3. One-parameter generalized logistic IRT model

According to Glogit models, μ(η) forms a cumulative distribution function for η, which can be interpreted as the probability of a correct answer in IRT. Building on the traditional difficulty and ability parameters in a one-parameter IRT model, we reintroduce two shape parameters related to the item factors, denoted as **λ**_*j*_ = (λ_1*j*_, λ_2*j*_). Consequently, we can deduce that the one-parameter generalized logistic model (1PGlogit) can be articulated as follows:


(7) $$\begin{array}{l} P\left(x_{ij}=1|\theta_{i},\beta_{j},\lambda_{j}\right)=\frac{\exp\left\{h_{\lambda_{j}}\left(\theta_{i}-\beta_{j}\right)\right\}}{1+\exp\left\{h_{\lambda_{j}}\left(\theta_{i}-\beta_{j}\right)\right\}}. \end{array}$$

Furthermore, when θ_*i*_ − β_*j*_ > 0 (which implies that $P\left(x_{ij}=1|\theta_{i},\beta_{j},\lambda_{j}\right)>\frac{1}{2}$),


(8) $$\begin{array}{l} h_{\lambda_{j}}\left(\theta_{i}-\beta_{j}\right)=\{\begin{array}{cc} -\frac{\log(1-\lambda_{1j}(\theta_{i}-\beta_{j}))}{\lambda_{1j}}, & \lambda_{1j}<\text{0,}\\ \theta_{i}-\beta_{j}, & \lambda_{1j}=\text{0}\\ \frac{\exp\left(\lambda_{1j}(\theta_{i}-\beta_{j})\right)-1}{\lambda_{1j}}, & \lambda_{1j}>\text{0}. \end{array}, \end{array}$$

When θ_*i*_ − β_*j*_ ≤ 0, which implies that ($P\left(x_{ij}=1|\theta_{i},\beta_{j},\lambda_{j}\right)\leq \frac{1}{2}$),


(9) $$\begin{array}{l} h_{\lambda_{j}}\left(\theta_{i}-\beta_{j}\right)=\{\begin{array}{cc} \frac{\log(1-\lambda_{2j}\left|\theta_{i}-\beta_{j}\right|)}{\lambda_{2j}}, & \lambda_{2j}<\text{0,}\\ \theta_{i}-\beta_{j}, & \lambda_{2j}=\text{0}\\ -\frac{\exp\left(\lambda_{2j}\left|\theta_{i}-\beta_{j}\right|\right)-1}{\lambda_{2j}}, & \lambda_{2j}>\text{0}. \end{array}, \end{array}$$

Specifically, when λ_1*j*_ = λ_2*j*_ = 0, the 1PGlogit model reduces to the Rasch model as shown in Equation (2); when λ_1*j*_ = λ_2*j*_ ≈ 0.165, the 1PGlogit model becomes the traditional 1PNO model in Equation (1). This applies when θ_*i*_ − β_*j*_ ≤ 0, we have


(10) $$\begin{array}{l} P\left(x_{ij}=1|\theta_{i},\beta_{j}\right)=\frac{\exp\left\{-\frac{\exp\left\{0.165|\theta_{i}-\beta_{j}|\right\}-1}{0.165}\right\}}{1+\exp\left\{-\frac{\exp\left\{0.165|\theta_{i}-\beta_{j}|\right\}-1}{0.165}\right\}}, \end{array}$$

when θ_*i*_ − β_*j*_ > 0, we have


(11) $$\begin{array}{l} P\left(x_{ij}=1|\theta_{i},\beta_{j}\right)=\frac{\exp\left\{\frac{\exp\left\{0.165\left(\theta_{i}-\beta_{j}\right)\right\}-1}{0.165}\right\}}{1+\exp\left\{\frac{\exp\left\{0.165\left(\theta_{i}-\beta_{j}\right)\right\}-1}{0.165}\right\}}, \end{array}$$

In fact, the CLLM model in Equation (3) is also a special case of the 1PGlogit model when the two shape parameters are restricted to λ_1*j*_ ≈ 0.62 and λ_2*j*_ ≈ −0.037. Specifically, when θ_*i*_ − β_*j*_ ≤ 0,


(12) $$\begin{array}{l} P\left(x_{ij}=1|\theta_{i},\beta_{j}\right)=\frac{\exp\left\{-\frac{\log\left\{1+0.037|\theta_{i}-\beta_{j}|\right\}}{0.037}\right\}}{1+\exp\left\{-\frac{\log\left\{1+0.037|\theta_{i}-\beta_{j}|\right\}}{0.037}\right\}}, \end{array}$$

when θ_*i*_ − β_*j*_ > 0,


(13) $$\begin{array}{l} P\left(x_{ij}=1|\theta_{i},\beta_{j}\right)=\frac{\exp\left\{\frac{\exp\left\{0.62\left(\theta_{i}-\beta_{j}\right)\right\}-1}{0.62}\right\}}{1+\exp\left\{\frac{\exp\left\{0.62\left(\theta_{i}-\beta_{j}\right)\right\}-1}{0.62}\right\}}, \end{array}$$

**Figure 1:**
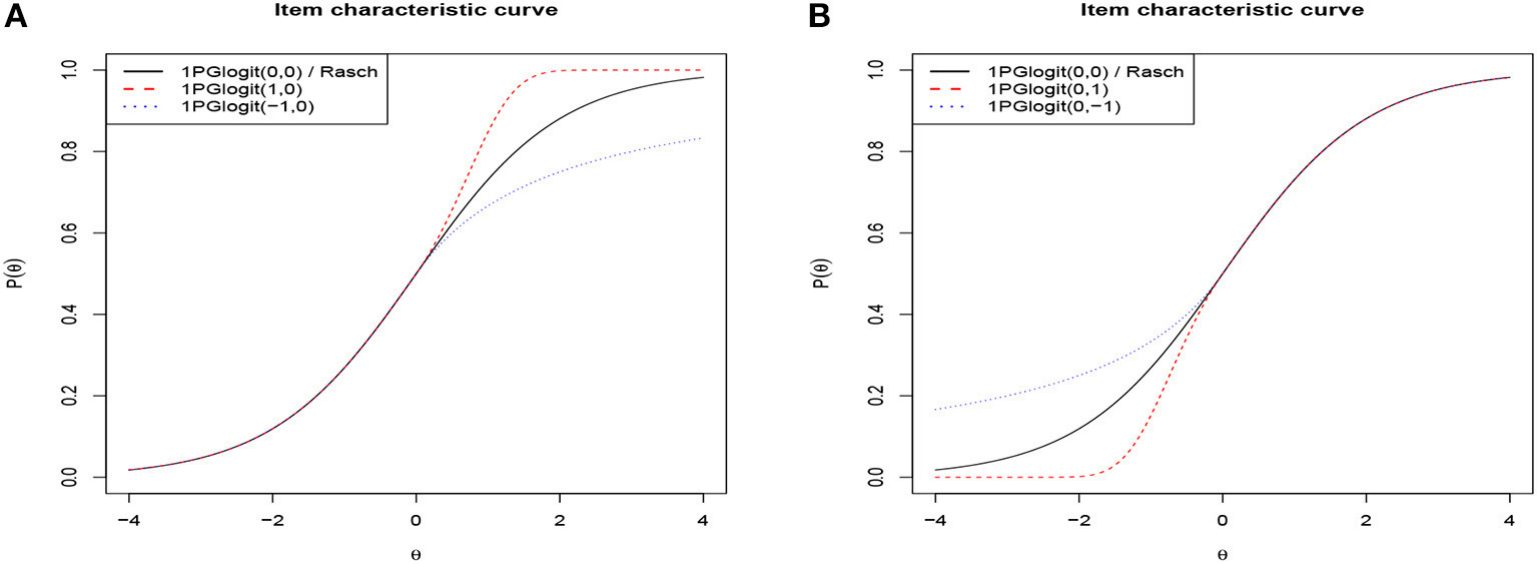
Item characteristic curves based on different the 1PGlogit models. **(A)** β_*j*_ = 0, λ_1*j*_ = 0, 1, −1 and λ_2*j*_ = 0. **(B)** β_*j*_ = 0, λ_1*j*_ = 0 and λ_2*j*_ = 0, 1, −1.

To intuitively explore 1PGlogit IRT models, we visualize the ICCs of 1PGlogit IRT models with different λ_1*j*_ and λ_2*j*_ in Figure 1, where the difficulty parameter *b* is set as 0. It can be observed from Figure 1 that parameters λ_1*j*_ and λ_2*j*_ control the convergence speed of the tail of 1PGlogit. The speed at which the tail of the ICC approaches 0 can be referred to as the “rate of convergence to the lower limit”. Similarly, the speed at which the ICC approaches 1 can be referred to as the “rate of convergence to the upper limit”. Specifically, Figure 1A shows that the parameter λ_1*j*_ controls the convergence speed to the upper asymptote, while Figure 1B shows that the parameter λ_2*j*_ controls the convergence speed to the lower asymptote. Common to both parameters is that the larger the value of λ_1*j*_ (λ_2*j*_), the faster the ICCs converge to the upper (lower) asymptote line. For instance, as shown in Figure 1A, when λ_1*j*_ = 1, the ICC of 1PGlogit(1, 0)has already converged to the upper asymptote *P*(θ) = 1 before θ = 2, while when λ_1*j*_ = 0, the ICC of 1PGlogit(0, 0) (i.e., Rasch model) just reaches the upper asymptote at θ = 4. However, when λ_1*j*_ = −1, the ICC of 1PGlogit(−1, 0) only converges to around *P*(θ) = 0.8 at θ = 4. The effect of the parameter λ_2*j*_ on the convergence of the ICC to the lower asymptote is similar to that of λ_1*j*_, which can be seen in Figure 1B.

**Figure 2:**
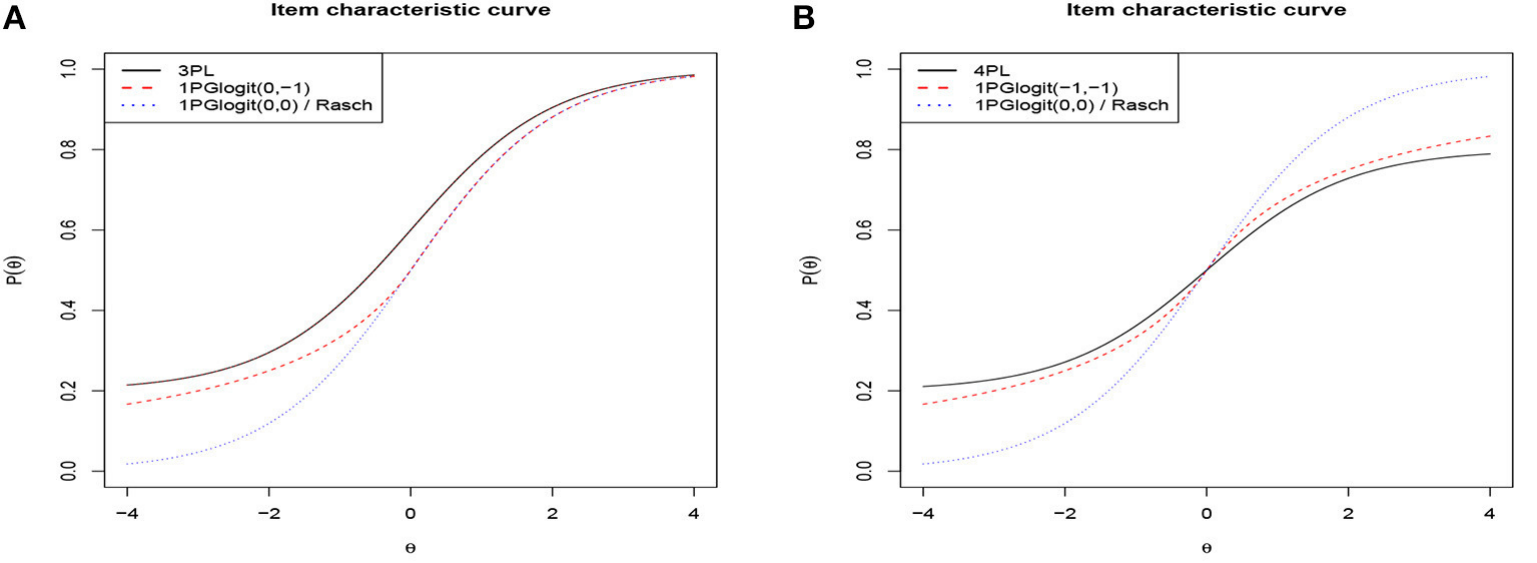
Item characteristic curves based on 3PL, 4PL, and 1PGlogit models. **(A)** 3PL model with α_*j*_ = 1, β_*j*_ = 0, *c*_*j*_ = 0.2. **(B)** 4PL model with α_*j*_ = 1, β_*j*_ = 0, *c*_*j*_ = 0.2, *d*_*j*_ = 0.8.

Based on the above analysis, it can be seen that the role of the parameter **λ**_*j*_ in 1PGlogit is somewhat analogous to the parameter *c* in the three-parameter logistic (3PL) model and the parameter *d* in the four-parameter logistic (4PL) model. As a result, we further compared the ICC of 1PGlogit with that of the 3PL model in Figure 2A and with the 4PL model in Figure 2B. Specifically, the expressions for the 3PL and 4PL models are as follows:


(14) $$\begin{array}{l} P\left(x_{ij}=1|\theta_{i},\alpha_{j},\beta_{j},c_{j}\right)=c_{j}+\left(1-c_{j}\right)\frac{\exp\left\{\alpha_{i}\left(\theta_{i}-\beta_{j}\right)\right\}}{1+\exp\left\{\alpha_{i}\left(\theta_{i}-\beta_{j}\right)\right\}}, \end{array}$$

and


(15) $$\begin{array}{l} P\left(x_{ij}=1|\theta_{i},\alpha_{j},\beta_{j},c_{j},d_{j}\right)=c_{j}+\left(d_{j}-c_{j}\right)\frac{\exp\left\{\alpha_{i}\left(\theta_{i}-\beta_{j}\right)\right\}}{1+\exp\left\{\alpha_{i}\left(\theta_{i}-\beta_{j}\right)\right\}}. \end{array}$$

In these models, α_*j*_ is the discrimination parameter, *c*_*j*_ is the lower asymptote parameter (which can be viewed as a guessing probability), and *d*_*j*_ is the upper asymptote parameter, where 1 − *d*_*j*_ can be considered as a slipping probability. For this analysis, we set α_*j*_ = 1, β_*j*_ = 0, *c*_*j*_ = 0.2, and *d*_*j*_ = 0.8. As demonstrated in Figure 2A, the 3PL model has an upper asymptote at *P*(θ) = 1 and a lower asymptote at *P*(θ) = 0.2, while the 1PGlogit(0, −1), with λ_1*j*_ = 0 and λ_2*j*_ = −1, displays an ICC similar to that of the 3PL model. In Figure 2B, the 4PL model exhibits an upper asymptote at *P*(θ) = 0.8 and a lower asymptote at *P*(θ) = 0.2. When λ_1*j*_ = −1 and λ_2*j*_ = −1, the 1PGlogit(−1, −1) shows an ICC comparable to the 4PL model. Hence, the parameter **λ**_*j*_ in 1PGlogit can be adjusted to represent the assumed guessing and slipping behaviors in the 3PL and 4PL models.

## 3. Bayesian estimation and model evaluations

In this study, we adopt the Bayesian statistical inference method to estimate the parameters in 1PGlogit IRT models. Let *P*_*ij*_ = *p*(*x*_*ij*_ = 1|β_*j*_, λ_1*j*_, λ_2*j*_, θ_*i*_), which is defined as shown in Equations (7)–(9). Thus, the likelihood function for the response of the *i*th examinee to the *j*th item can be written as:


(16) $$\begin{array}{l} p\left(x_{ij}|\beta_{j},\lambda_{1j},\lambda_{2j},\theta_{i}\right)=P_{ij}^{x_{ij}}{\left(1-P_{ij}\right)}^{1-x_{ij}}. \end{array}$$

Let **x** = (*x*_*i*_, ⋯  , *x*_*N*_), **β** = (β_1_, ⋯  , β_*J*_), **λ_1_** = (λ_11_, ⋯  , λ_1*J*_), **λ_2_** = (λ_21_, ⋯  , λ_2*J*_), **θ** = (θ_1_, ⋯  , θ_*N*_). Then the joint posterior distribution of parameters **β**, **λ_1_**, **λ_2_**, and **θ** can be derived as:


(17) $$\begin{array}{l} \begin{array}{c} p\left(\beta ,\lambda_{1},\lambda_{2},\theta |x\right)=p\left(x|\beta ,\lambda_{1},\lambda_{2},\theta \right)p\left(\beta \right)p\left(\lambda_{1}\right)p\left(\lambda_{2}\right)p\left(\theta \right),\\ =\underset{\text{Likelihood function}}{\underset{︸}{\left\{\overset{N}{\underset{i=1}{\prod }}\overset{J}{\underset{j=1}{\prod }}p\left(x_{ij}|\beta_{j},\lambda_{1j},\lambda_{2j},\theta_{i}\right)\right\}}}\underset{\text{Prior distributions}}{\underset{︸}{\left\{\overset{J}{\underset{j=1}{\prod }}p\left(\beta_{j}\right)p\left(\lambda_{1j}\right)p\left(\lambda_{2j}\right)\right\}\left\{\overset{N}{\underset{i=1}{\prod }}p\left(\theta_{i}\right)\right\}}}. \end{array} \end{array}$$

### 3.1. Prior distributions

According to Chen et al. (2002) and Chen et al. (1999), it is necessary to constrain the parameters λ_1*j*_ and λ_2*j*_ to be greater than −1 to ensure a proper posterior distribution. Therefore, the priors for λ_1*j*_ and λ_2*j*_ should be truncated at −1. The parameters β_*j*_ and θ_*i*_ are assumed to follow different normal prior distributions, while λ_1*j*_ and λ_2*j*_ are assumed to follow a truncated normal prior distribution. Overall, the priors for the parameters are set as follows:


(18) $$\begin{array}{l} \begin{array}{c} \beta_{j}\sim \text{N}\left(0,\sigma_{\beta }^{2}\right),\\ \lambda_{1j}\sim \text{N}\left(0,\sigma_{\lambda }^{2}\right)I\left(-1,\infty \right),\\ \lambda_{2j}\sim \text{N}\left(0,\sigma_{\lambda }^{2}\right)I\left(-1,\infty \right),\\ \theta_{i}\sim \text{N}\left(0,1\right),\\ \sigma_{\beta }\sim \text{Cauchy}\left(0,5\right)I\left(0,\infty \right),\\ \sigma_{\lambda }\sim \text{Cauchy}\left(0,5\right)I\left(0,\infty \right), \end{array} \end{array}$$

where I(a,b) implies that the parameter is constrained within the interval (*a, b*).

### 3.2. Stan software

In this paper, we employ the MCMC method for parameter estimation. Currently, there are various software options available for implementing the MCMC algorithm, such as WinBUGS (Lunn et al., 2000), OpenBUGS (Spiegelhalter et al., 2010), and JAGS (Plummer, 2003). However, In the subsequent research, we utilize the Stan software (Stan Development Team, 2019), which is based on the Hamiltonian Monte Carlo (HMC) algorithm (Neal, 2011) and the no-U-turn sampler (NUTS) (Hoffman and Gelman, 2014). HMC efficiently explores posteriors in models and is often faster than the Gibbs method (Geman and Geman, 1984) and the Metropolis algorithm (Metropolis et al., 1953), while NUTS further improves efficiency. Additionally, Stan provides interfaces with data analysis languages such as R, Python, Matlab, etc., making it convenient for our use. To implement the Stan program, we specifically utilize the R package **rstan**, which interfaces with Stan in R (R Core Team, 2019). The Stan code employed for parameter estimation in this study, along with the actual data, can be found at the following URL: https://github.com/X-Wang777/-A-Generalized-One-Parameter-IRT. Furthermore, Luo and Jiao (2018) offer a detailed tutorial on utilizing Stan for estimating various IRT models.

### 3.3. Criteria for assessing parameter estimation accuracy

In this research, we will use four criteria for assessing the accuracy of parameter estimation. They are Bias, RMSE (Root Mean Squared Error), SE (Standard Error), and SD (Standard Deviation). Assuming the parameter of interest is β_*j*_, the evaluation criteria based on the β_*j*_ parameter are defined as follows:


$$\begin{array}{c} \text{Bias}\left(\beta_{j}\right)=\frac{1}{R}\overset{R}{\underset{r=1}{\sum }}\left({\hat{\beta }}_{j}^{\left(r\right)}-\beta_{j}\right),\\ \text{RMSE}\left(\beta_{j}\right)=\sqrt{\frac{1}{R}\overset{R}{\underset{r=1}{\sum }}{\left({\hat{\beta }}_{j}^{\left(r\right)}-\beta_{j}\right)}^{2}},\\ \text{SE}\left(\beta_{j}\right)=\sqrt{\frac{1}{R}\overset{R}{\underset{r=1}{\sum }}{\left({\hat{\beta }}_{j}^{\left(r\right)}-\frac{1}{R}\overset{R}{\underset{l=1}{\sum }}{\hat{\beta }}_{j}^{\left(l\right)}\right)}^{2}},\\ \text{SD}\left(\beta_{j}\right)=\frac{1}{R}\overset{R}{\underset{r=1}{\sum }}SD^{\left(r\right)}\left(\beta_{j}\right). \end{array}$$

where *R* denotes the number of replications and ${\hat{\beta }}_{j}^{\left(r\right)}$ is the estimate of β_*j*_ in the *r*th replication, and ${\text{SD}}^{\left(r\right)}\left(\beta_{j}\right)$ is the posterior standard deviation of β_*j*_ in the *r*th replication. Thus, we are able to calculate the average values for the four accuracy assessment indicators based on all items. That is,


Average Bias(β)=1J×R∑j=1J∑r=1R(^j(r)-βj),Average RMSE(β)=1J∑j=1J1R∑r=1R(β^j(r)-βj)2,Average SE(β)=1J∑j=1J1R∑r=1R(β^j(r)-1R∑l=1Rβ^j(l))2,Average SD(β)=1J×R∑j=1J∑r=1RSD(r)(βj).

### 3.4. Bayesian model assessment

The following four model selection criteria will be used in this paper to evaluate the goodness of model fit: (1) DIC, (2) Logarithm of the pseudomarginal Likelihood (LPML; Geisser and Eddy, 1979; Ibrahim et al., 2001), (3) Widely applicable information criterion (WAIC; Watanabe and Opper, 2010), and (4) Leave-one-out cross-validation (LOO; Vehtari et al., 2017). In addition, the last two information criteria are calculated based on the R package **loo** (Vehtari et al., 2017).

## 4. Simulation studies

### 4.1. Simulation 1

In this simulation study, our aim is to assess the accuracy of parameter estimation for various one-parameter symmetric and asymmetric IRT models implemented using the Stan software. The following four models will be considered: (1) 1PGlogit(λ_1*j*_, λ_2*j*_), *j* = 1, 2, ..., *J*; (2) Rasch (1PGlogit(0, 0)); (3) 1PNO (1PGlogit(0.165, 0.165)); and (4) CLLM (1PGlogit(0.62, −0.037)).

#### 4.1.1. Simulation designs

The true values of the parameters are generated following this formulation: θ~*N*(0, 1), *b* ~ *N*(0, 1). For the 1PGlogit(λ_1*j*_, λ_2*j*_) model, the true values of (λ_1*j*_, λ_2*j*_) are generated from the distribution $\lambda_{1j}\sim N\left(0,0.5^{2}\right)I\left(-1,+\infty \right)$, $\lambda_{2j}\sim N\left(0,0.5^{2}\right)I\left(-1,+\infty \right)$. Meanwhile, λ_1*j*_ is fixed at 0, 0.165, and 0.62 for the Rasch, 1PNO, and CLLM models, respectively, while λ_2*j*_ is fixed at 0, 0.165, and –0.037, respectively. The manipulated factors include sample size (i.e., the number of students) *N* = 1, 000, 2,000, and item length *J* = 20, 40. Thus, there are four simulation conditions for each model, and each simulation condition was replicated 50 times. We set four chains in each simulation, each executing 3,000 iterations, and the burn-in period is 2,000 iterations.

#### 4.1.2. Convergence diagnosis

**Figure 3:**
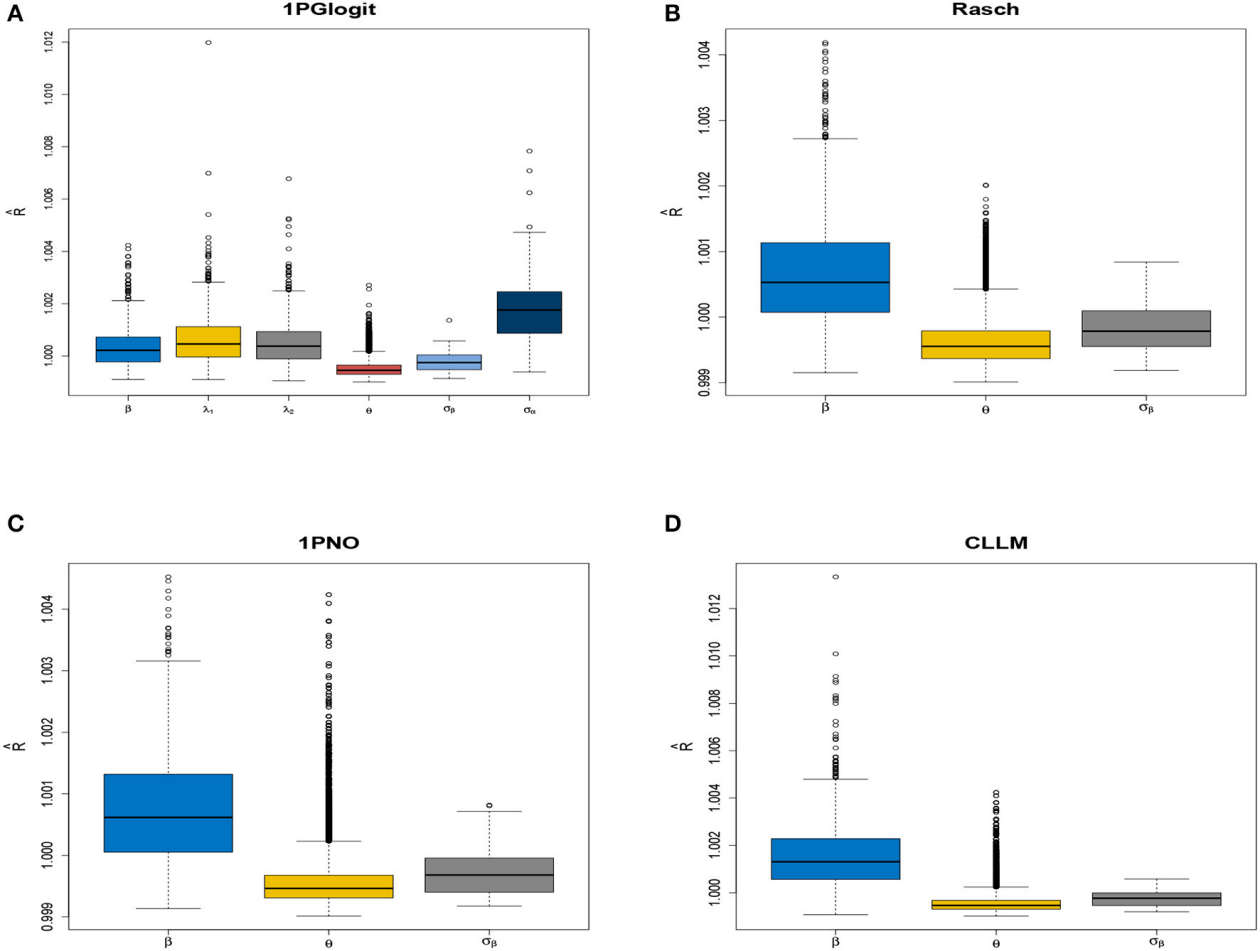
Boxplot of parameter $\hat{R}$ in four models under *N* = 1, 000 and *J* = 20 conditions in simulation 1. **(A)** 1PGlogit model. **(B)** Rasch model. **(C)** 1PNO model. **(D)** CLLM.

**Figure 4:**
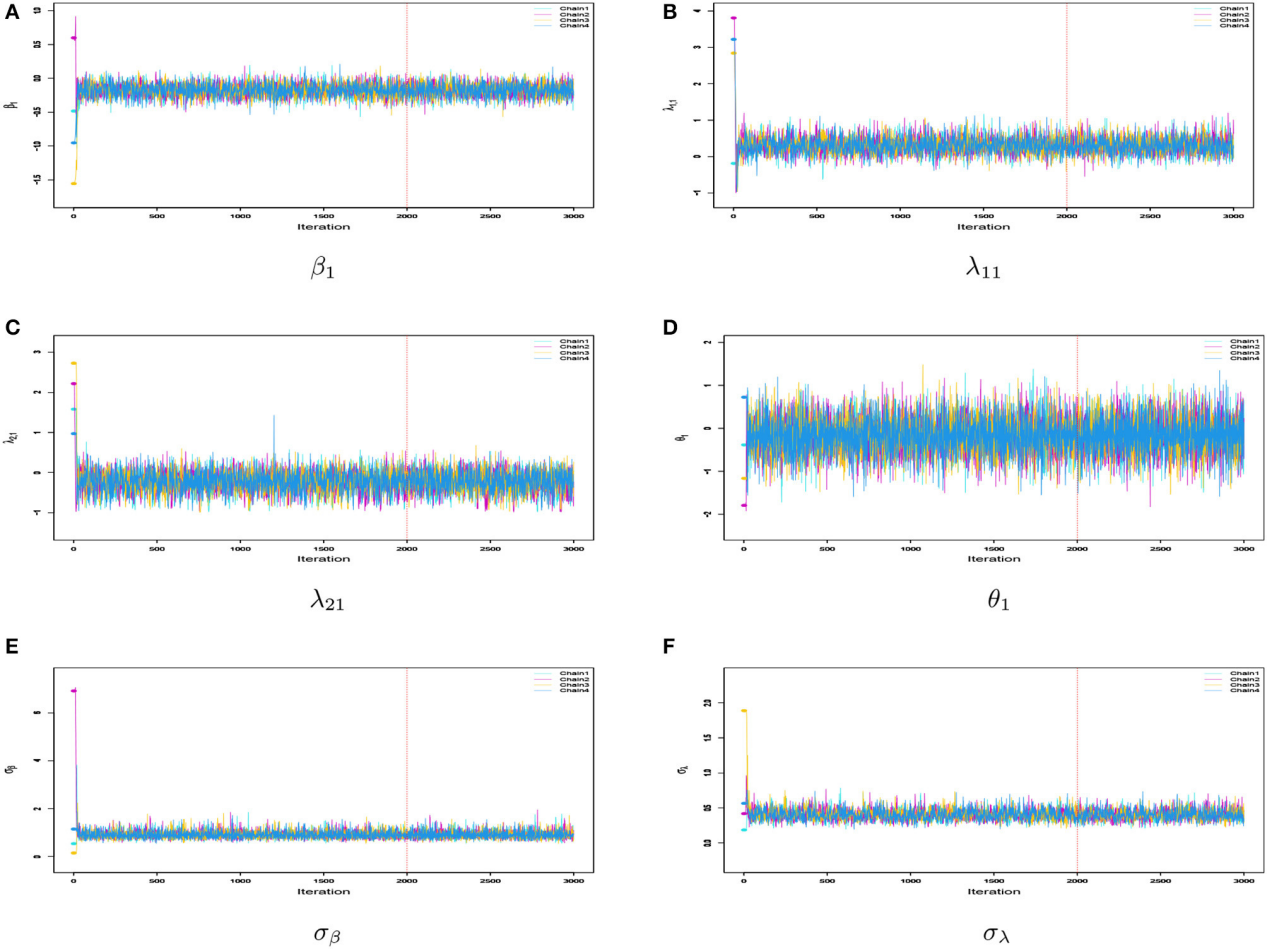
**(A–F)** Sampling trace plots of parameters β_1_, λ_11_, λ_21_, θ_1_, σ_β_, and σ_**λ**_ in four Markov chains for 1PGlogit model under *N* = 1, 000 and *J* = 20 conditions in simulation 1.

Firstly, we examined the convergence of the MCMC procedure implemented in **rstan**. As an example, we considered the case with *N* = 1, 000 and *J* = 20. The potential scale reduction factor (PSRF; also known as $\hat{R}$, Brooks and Gelman, 1998) values of the parameters in each model are shown in Figure 3, which presents a boxplot of the $\hat{R}$ values for all difficulty parameters across 50 repeated simulations. It can be observed that the $\hat{R}$ for all parameters in each model is close to 1 and less than 1.05, indicating that all parameters have converged. In addition, we selected the parameters for the first item, namely β_1_, λ_11_, λ_21_, as well as the latent trait of the first student θ_1_ and the standard deviations σ_β_, σ_**λ**_. We plotted the MCMC traces of these parameters across the four chains in Figure 4. The red vertical line represents the burn-in value and the colored circles represent the initial values. From the trace plots, it is apparent that all parameters reached stationarity before the burn-in period, which further validates that the convergence is assured when using the Stan software for parameter estimation.

#### 4.1.3. Analysis of parameter estimation accuracy

In this study, we examine the accuracy of the estimation for the item parameters and latent trait parameters of each model. We computed the average bias, MSE, SE, and SD for each parameter, which are presented in Table 1. By examining the results in the table, we draw the following conclusions: First, the estimation appears unbiased, as reflected by the minimal and close-to-zero bias of all parameters. Second, our estimation exhibits large sample properties, meaning the precision of parameter estimation improves as the number of students increases for item parameters, and as the number of items increases for ability parameters. For instance, in the 1PGlogit model, as the sample size increases from *N*=1,000 to *N*=2,000, the MSE, SE, and SD of item parameters **β**, **λ_1_**, **λ_2_** decrease. Similarly, when increasing from *J*=20 to *J*=40, the MSE, SE, and SD of **θ** decrease as well. Similar conclusions hold true in the Rasch, 1PNO, and CLLM models. Moreover, we observed that the estimation precision of latent trait parameters **θ** is not as robust as that of difficulty parameters **β** across all models. This can be attributed to the limited number of items (only 20 or 40 items). Specifically, in the 1PGlogit model, the estimation precision of **λ** is also poorer than that of **β**, and we speculate that this may be due to the interaction between **λ** and **θ** affecting the estimation precision.

**Table 1:** Evaluating the accuracy of parameter estimation for various models and simulation conditions in simulation study 1.

		***N*** = 1, 000				***N*** = 2, 000			
		*Bias*	*MSE*	*SE*	*SD*	*Bias*	*MSE*	*SE*	*SD*
*J* = 20									
1PGlogit	**β** **λ_1_** **λ_2_** **θ**	–0.0310	0.0102	0.0869	0.1009	–0.0361	0.0063	0.0642	0.0729
		–0.0452	0.0972	0.1381	0.2246	0.0123	0.0566	0.1359	0.1945
		–0.0406	0.0785	0.1513	0.2530	–0.0284	0.0508	0.1395	0.2141
		–0.0281	0.1869	0.3825	0.4416	0.0389	0.1907	0.3803	0.4311
Rasch	**β** **θ**	0.0329	0.0067	0.0728	0.0792	–0.0033	0.0027	0.0498	0.0563
		0.0337	0.2058	0.3988	0.4583	–0.0024	0.2168	0.3993	0.4597
1PNO	**β** **θ**	0.0287	0.0051	0.0642	0.0740	0.0166	0.0025	0.0463	0.0526
		0.0344	0.1879	0.3807	0.4293	0.0148	0.1857	0.3809	0.4293
CLLM	**β** **θ**	–0.0146	0.0046	0.0639	0.0710	-0.0095	0.0020	0.0428	0.0505
		–0.0103	0.1701	0.3616	0.4073	–0.0058	0.1697	0.3608	0.4082
*J* = 40									
1PGlogit	**β** **λ_1_** **λ_2_** **θ**	–0.0434	0.0138	0.0936	0.1082	0.0064	0.0067	0.0728	0.0798
		–0.0383	0.0912	0.1503	0.2449	–0.0201	0.0631	0.1344	0.2079
		0.0318	0.0938	0.1755	0.2574	–0.0167	0.0684	0.1575	0.2111
		–0.0280	0.1166	0.3102	0.3378	–0.0091	0.1135	0.3104	0.3373
Rasch	**β** **θ**	0.0296	0.0061	0.0713	0.0792	0.0046	0.0027	0.0507	0.0563
		0.0331	0.1197	0.3201	0.3462	0.0048	0.1223	0.3190	0.3466
1PNO	**β** **θ**	–0.0049	0.0045	0.0658	0.0739	0.0110	0.0024	0.0471	0.0524
		–0.0049	0.1036	0.2976	0.3209	0.0117	0.1029	0.2981	0.3198
CLLM	**β** **θ**	–0.0118	0.0047	0.0657	0.0726	–0.0078	0.0023	0.0456	0.0516
		–0.0104	0.0943	0.2828	0.3054	–0.0059	0.0954	0.2843	0.3057

The terms Bias, MSE, SD, and SE denote the average bias, mean square error (MSE), standard deviation (SD), and standard error (SE) of the parameters, respectively.

### 4.2. Simulation 2

In this simulation study, our aim is to assess the model fit of traditional symmetric IRT models, asymmetric IRT model, and the Glogit IRT models under the framework of the one-parameter IRT.

We consider a sample size of *N* = 1, 000 individuals, with the test length fixed at 20. Item responses are generated within the framework of a one-parameter IRT model. We consider four item response models: (1) 1PGlogit(λ_1*j*_, λ_2*j*_), *j* = 1, 2, ..., *J*; (2) Rasch (1PGlogit(0, 0)); (3) 1PNO (1PGlogit(0.165, 0.165)); and (4) CLLM (1PGlogit(0.62, −0.037)). Therefore, we evaluate the model fitting in the following four cases.

- Case 1: True model: 1PGlogit(λ_1*j*_, λ_2*j*_) v.s. Fitted model: 1PGlogit(λ_1*j*_, λ_2*j*_), Rasch, 1PNO, and CLLM;
- Case 2: True model: Rasch v.s. Fitted model: 1PGlogit(λ_1*j*_, λ_2*j*_), Rasch, 1PNO, and CLLM;
- Case 3: True model: 1PNO v.s. Fitted model: 1PGlogit(λ_1*j*_, λ_2*j*_), Rasch, 1PNO, and CLLM;
- Case 4: True model: CLMM v.s. Fitted model: 1PGlogit(λ_1*j*_, λ_2*j*_), Rasch, 1PNO, and CLLM.

**Figure 5:**
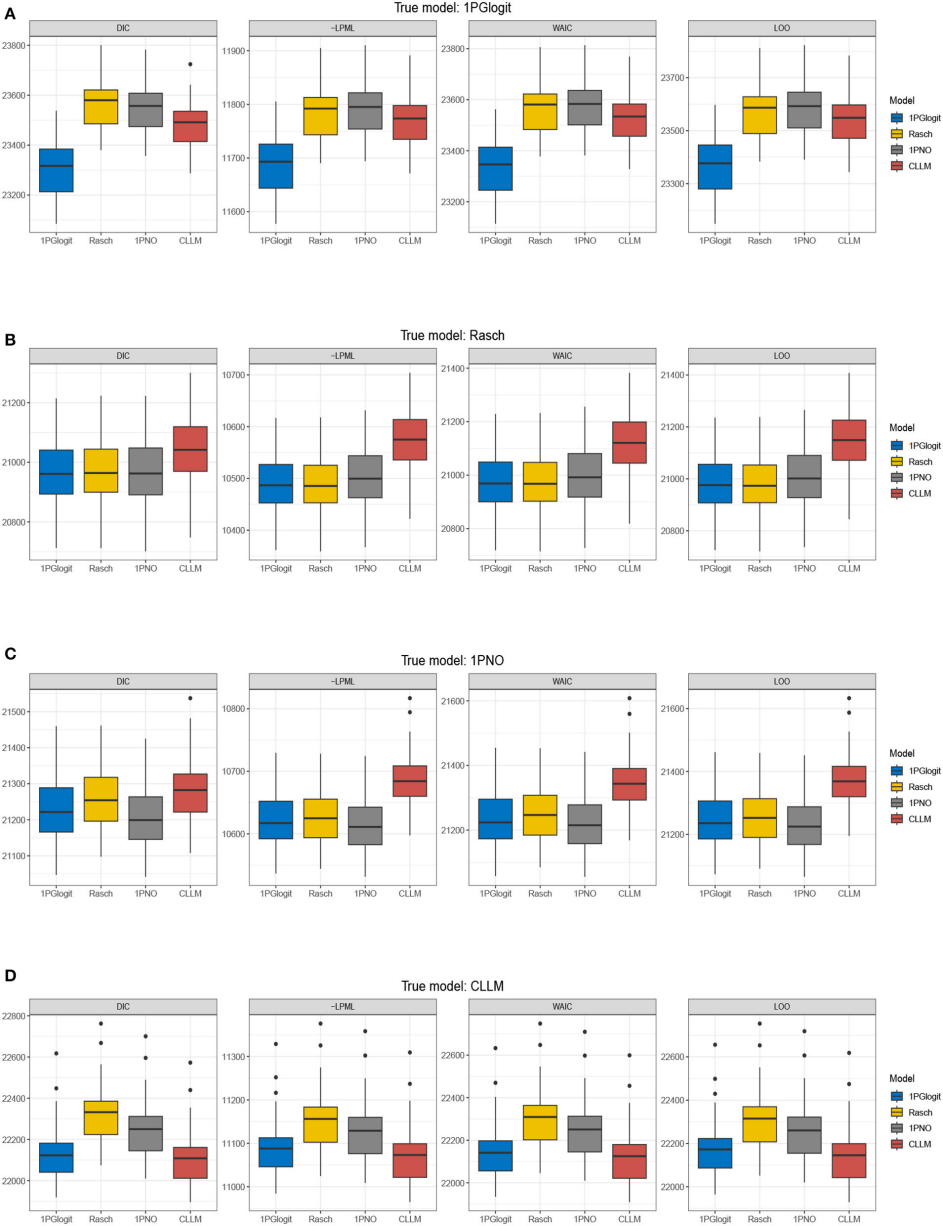
Boxplots of DIC, –LPML, WAIC, and LOO for 1PGlogit, Rasch, 1PNO, and CLLM models in simulation 2. **(A)** True model: 1PGlogit model. **(B)** True model: Rasch model. **(C)** True model: 1PNO model. **(D)** True model: CLLM.

The true values and prior distributions for the parameters are specified in the same way as in simulation 1. To implement the MCMC sampling algorithm, chains of length 3,000 are chosen, with an initial burn-in period of 2,000. The results of the Bayesian model assessment, based on 50 replications, are shown in Table 2. It is worth noting that the reported results of DIC, LPML, WAIC, and LOO are based on the average of these 50 replications. The corresponding boxplots of the four Bayesian model assessment indexes is shown in Figure 5. Additionally, we have compiled the number of times each model was selected as the best or second-best model in Table 3.

**Table 2:** Comparing the DIC, LPML, WAIC, and LOO values for 1PGlogit, Rasch, 1PNO, and CLLM models in simulation 2.

**Fitted model**	**DIC**	**LPML**	**WAIC**	**LOO**
**True model: 1PGlogit**				
1PGlogit Rasch 1PNO CLLM	**23306.55**	**–11686.98**	**23335.52**	**23367.22**
	23559.69	–11781.79	23560.16	23566.15
	23546.30	–11790.91	23574.84	23584.28
	23476.40	–11767.11	23520.99	23535.90
**True model: Rasch**				
1PGlogit Rasch 1PNO CLLM	20965.74	–10489.26	**20974.06**	20980.80
	20970.16	**–10488.71**	20974.30	**20979.91**
	**20964.79**	–10500.66	20994.16	21003.703
	21043.82	–10575.56	21123.53	21150.62
**True model: 1PNO**				
1PGlogit Rasch 1PNO CLLM	22122.17	–10618.52	21226.77	21238.39
	21250.88	–10622.21	21241.22	21246.88
	**21201.76**	**–10611.60**	**21215.99**	**21225.56**
	21278.45	–10684.17	21342.14	21367.91
**True model: CLLM**				
1PGlogit Rasch 1PNO CLLM	22131.63	–11091.62	22148.53	22178.66
	22330.45	–11155.46	22307.58	22313.39
	22252.20	–11129.47	22251.93	22261.30
	**22111.62**	**–11073.75**	**22127.46**	**22147.63**

The bold values represent the minimum values of the corresponding model selection criteria across all candidate models.

**Table 3:** Number of times selected as the best model and the second-best model based on DIC, LPML, WAIC, and LOO in simulation 2.

	**Times of selected as the best model**				**Times of selected as the second-best model**			
	**1PGlogit**	**Rasch**	**1PNO**	**CLLM**	**1PGlogit**	**Rasch**	**1PNO**	**CLLM**
**True model: 1PGlogit**								
DIC LPML WAIC LOO	50	0	0	0	0	0	0	50
	50	0	0	0	0	2	0	48
	50	0	0	0	0	0	0	50
	50	0	0	0	0	2	0	48
**True model: Rasch**								
DIC LPML WAIC LOO	20	2	28	0	25	21	4	0
	13	36	1	0	36	14	0	0
	24	25	1	0	25	24	1	0
	15	34	1	0	34	16	0	0
**True model: 1PNO**								
DIC LPML WAIC LOO	2	0	48	0	47	2	1	0
	2	0	48	0	37	12	1	0
	3	0	47	0	42	5	3	0
	3	0	47	0	37	1	1	2
**True model: CLLM**								
DIC LPML WAIC LOO	4	0	0	46	46	0	0	4
	0	0	0	50	0	0	0	50
	4	0	0	46	46	0	0	4
	0	0	0	50	0	0	0	50

According to Tables 2, 3, when the true model is a 1PGlogit model, the 1PGlogit model is consistently chosen as the optimal model for data fitting based on the average values of the four model evaluation criteria, compared to the other three competing models. The second-best model is mostly the asymmetric CLLM, except for two instances where the Rasch model is selected for LPML and LOO criteria. When the true model is the CLLM model, the evaluation results are very similar to the case where the true model is the 1PGlogit model. With only a few exceptions, the CLLM model is chosen as the optimal model for almost all evaluation indicators, and the 1PGlogit model is chosen as the second-best model. Additionally, from Table 2 and Figure 5, we can observe that the fitting results of the 1PGlogit model are not significantly different from that of the CLLM model. In fact, 1PGlogit model has been selected four times as the best model using DIC and WAIC. However, the fitting results of the other two symmetric models, Rasch and 1PNO, are noticeably worse compared with that of the CLLM and 1PGlogit models. Interestingly, when the true model is the Rasch model, we observe that the fitting results of the 1PGlogit and 1PNO models are highly similar to those of the Rasch model. In terms of average DIC value, the 1PGlogit and 1PNO models even perform better and are often chosen as the best models. The Rasch model has only a very slight advantage over the 1PGlogit model in LPML and LOO, and in many cases, the 1PGlogit model is selected as the true model. The difference between 1PGlogit model and Rasch model, based on the four model evaluation criteria, is very small and less than 1. The fitting results of the 1PNO model are slightly worse than that of 1PGlogit and Rasch models based on LPML, WAIC, and LOO criteria, and the performance of the CLLM is the worst in all four evaluation criteria. In the case where the 1PNO model is the true model, we also observe that the performance of the CLLM is consistently the worst. While the 1PNO model slightly outperforms the 1PGlogit model across all model evaluation criteria, the 1PGlogit model still provides a good fit and has been selected as the best fitting model several times based on these model evaluation criteria.

**Figure 6:**
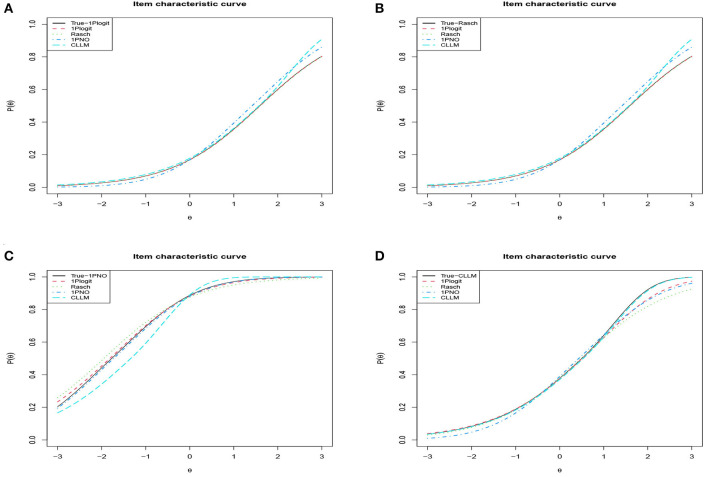
Analyzing the degree of fit for ICCs across different true models and fitting models in simulation 2. **(A)** True model: 1PGlogit with β_1_ = −0.5734, λ_11_ = 1, λ_21_ = 1. **(B)** True model: Rasch (1PGlogit with β_1_ = 1.5891, λ_11_ = 0, λ_21_ = 0). **(C)** True model: 1PNO (1PGlogit with β_1_ = −1.7712, λ_11_ = 0.165, λ_21_ = 0.165). **(D)** True model: CLLM (1PGlogit with β_1_ = 0.5036, λ_11_ = 0.62, λ_21_ = −0.037).

Additionally, we chose the first item from four simulation conditions, respectively, and plotted their true ICCs against the four fitted ICCs for comparison in Figure 6. The true ICC is represented by the black line, while the red line illustrates the ICC fitted using 1PGlogit model. It can be noted that regardless of the true model type, our 1PGlogit model can provide an excellent fit, especially when the Rasch model and 1PNO model serve as the true model, the ICC fitted by 1PGlogit model almost coincides with the true ICC curve. In summary, 1PGlogit model proves to be a versatile generalized model that fits several widely used one-parameter IRT models effectively.

### 4.3. Simulation 3

In our previous discussion, we noted that the two shape parameters in the proposed 1PGlogit model can control whether the ICC has a heavy or light tail, playing a role similar to the lower asymptote parameter in the three-parameter IRT models, and the upper asymptote parameter in the more generalized four-parameter IRT models. In this simulation study, we focus on comparing the fit superiority of the 1PGlogit model with the traditional 3PL and 4PL models.

We consider a sample size of *N* = 1, 000 individuals, with the test length fixed at 20. Item responses are generated from the 3PL model and 4PL model. Therefore, we evaluate the model fitting in the following two cases.

- Case 1: True model: 3PL v.s. Fitted model: 1PGlogit(λ_1*j*_, λ_2*j*_), Rasch, 1PNO, CLLM, and 3PL;
- Case 2: True model: 4PL v.s. Fitted model: 1PGlogit(λ_1*j*_, λ_2*j*_), Rasch, 1PNO, and CLLM, and 4PL.

The true values of parameters in the 3PL and 4PL models are generated as follows: α_*j*_ ~ U(0.5, 2), β_*j*_ ~ N(0, 1), *c*_*j*_ ~ Beta(5, 17) and *d*_*j*_ ~ Beta(17, 5) (*d*_*j*_ = 1 for 3PL model). The prior distribution of parameters in the 1PGlogit model, Rasch model, 1PNO model, and CLLM are generated the same as in simulation 1. Moreover, we wish to clarify the prior distributions setting for the parameters in the 3PL/4PL models: logα_*j*_ ~ N(0, 1), $\beta_{j}\sim \text{N}\left(0,\sigma_{\beta }^{2}\right)$, *c*_*j*_ ~ U(0, 0.5), *d*_*j*_ ~ U(0, 0.5) (in 4PL model), and σ_β_ ~ Cauchy(0, 5). To implement the MCMC sampling algorithm, chains of length 5,000 are chosen, with an initial burn-in period of 4,000.

**Figure 7:**
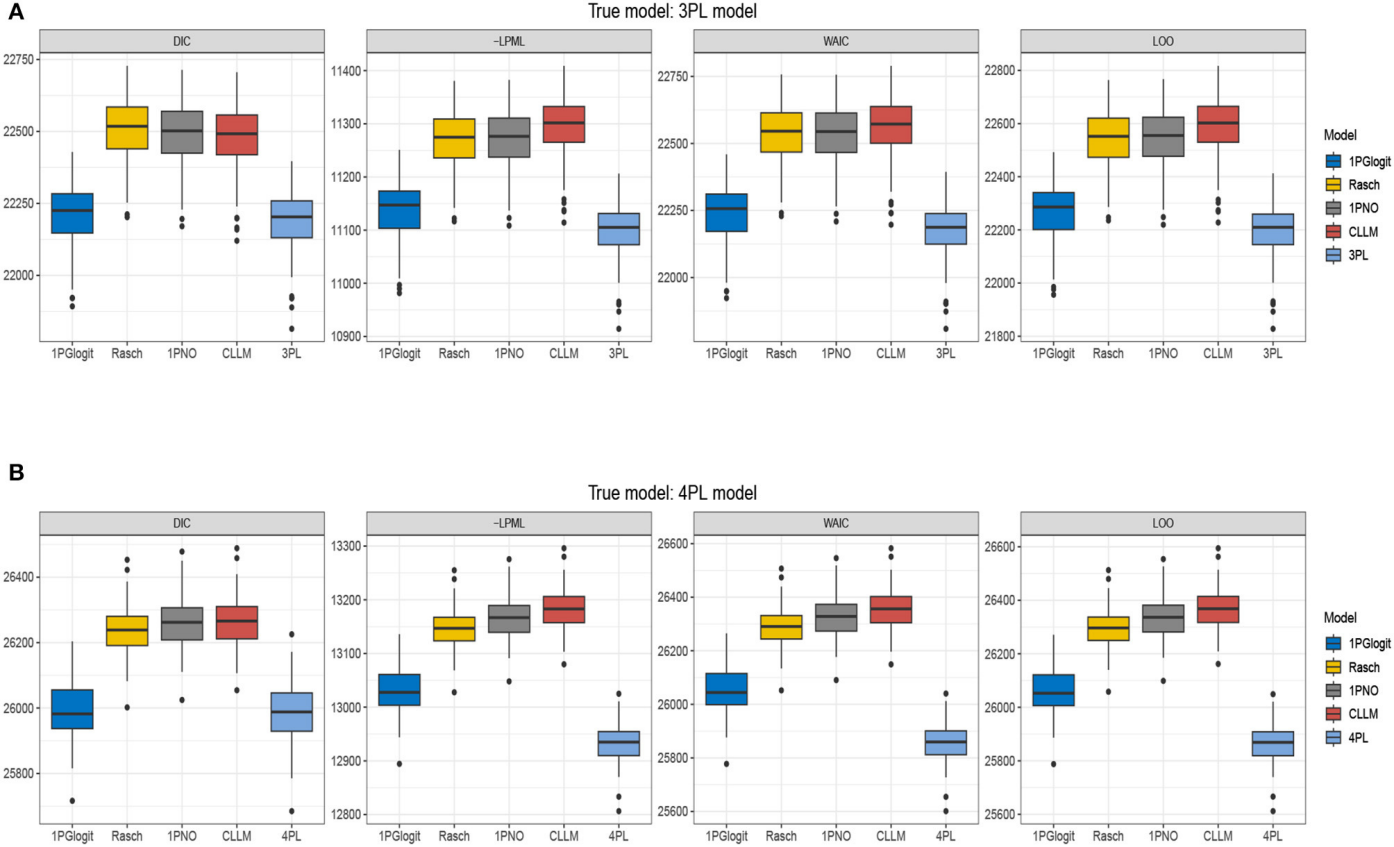
Boxplots of DIC, –LPML, WAIC, and LOO for 1PGlogit, Rasch, 1PNO, CLLM, 3PL, and 4PL models in simulation 3. **(A)** True model: 3PL model. **(B)** True model: 4PL model.

**Figure 8:**
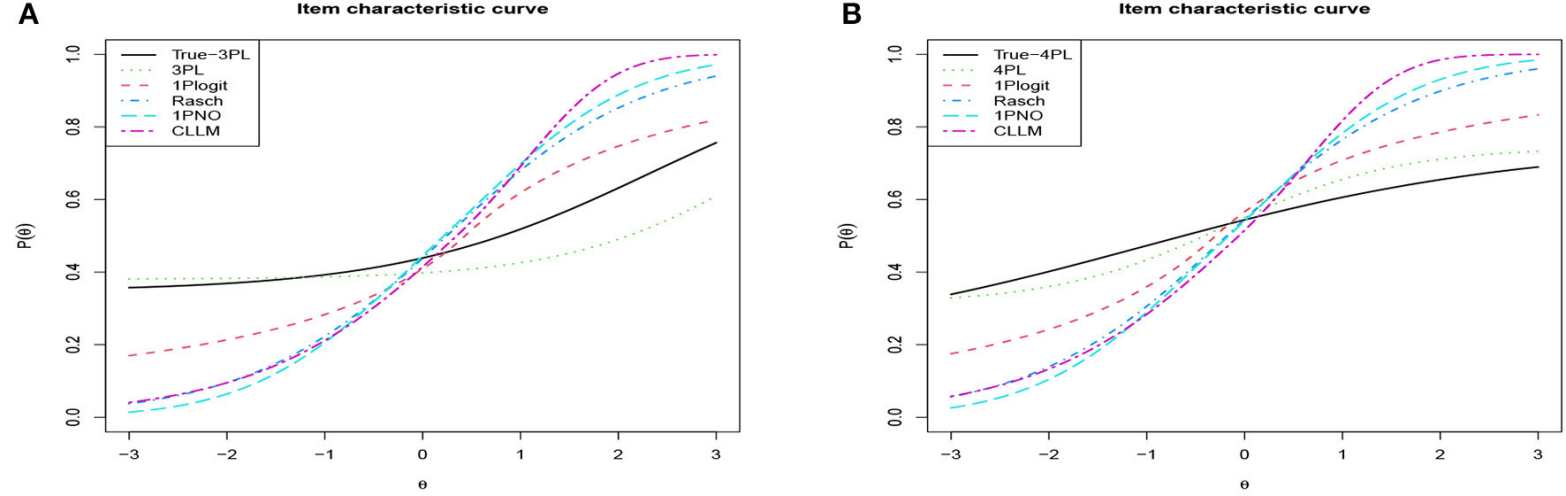
Analyzing the degree of fit for ICCs across different true models and fitting models in simulation 3. **(A)** True model: 3PL model with α_1_ = 0.7766, β_1_ = 2.3315, *c*_1_ = 0.3470. **(B)** True model: 4PL model with α_1_ = 0.5167, β_1_ = −1.0322, *c*_1_ = 0.1894, *d*_1_ = 0.7518.

In Table 4, we present the DIC, LPML, WAIC, and LOO values for each model. Figure 7 depicts the boxplots of these four model selection criteria across 50 replications. Additionally, Table 5 summarizes the instances where each model was selected as the best or second best fitting model across the 50 replications. The results indicate that when the true model is the 3PL model, the average values of –LPML, WAIC, and LOO for the 3PL model are the lowest among all models under consideration. In all 50 replications, these evaluation criteria identify the true 3PL model as the best model. For the second-best model selection, apart from LOO (which chose the Rasch model once), all other criteria consistently select the 1PGlogit model. Although the average DIC value for the 3PL model is the lowest, it differs from the other three criteria. In 12 out of 50 replications, the 1PGlogit model is selected as the best model, and in 38 replications, it's chosen as the second-best model. These findings suggest that our flexible 1PGlogit model can effectively fit the 3PL model. Considering the values of various model selection criteria and the boxplot results, the fitting performance of the 1PGlogit model is significantly superior to other one-parameter models. To further illustrate this, we plotted the ICC of the first item for the true 3PL model, as well as ICC curves fitted by the five different models in Figure 8. The plots reveal that, aside from the fitted 3PL model, our 1PGlogit model shows the best fit with the true ICC, regardless of item difficulty. In the 3PL model, the assumed guessing behavior causes the lower asymptote of its ICC to be above zero. Our 1PGlogit model can account for this phenomenon through the parameter **λ**, suggesting that our model can also interpret the assumed guessing behavior inherent in the 3PL model.

**Table 4:** Comparing the DIC, LPML, WAIC, and LOO values for 1PGlogit, Rasch, 1PNO, CLLM, 3PL, and 4PL models in simulation 3.

**Fitted model**	**DIC**	**LPML**	**WAIC**	**LOO**
**True model: 3PL model**				
1PGlogit Rasch 1PNO CLLM 3PL	22205.12	–11136.05	22234.30	22265.62
	22504.53	–11268.28	22532.98	22533.96
	22486.74	–11268.22	22528.35	22538.81
	22469.30	–11290.41	22550.59	22579.95
	**22186.26**	**–11095.16**	**22169.37**	**22189.71**
**True model: 4PL model**				
1PGlogit Rasch 1PNO CLLM 4PL	25991.13	–13031.57	26054.59	26062.20
	26234.96	–13144.81	26286.36	26292.44
	26257.85	–13164.60	26323.77	26332.01
	26266.45	–13183.73	26357.70	26369.97
	**25985.41**	**–12933.86**	**25857.94**	**25866.80**

The bold values represent the minimum values of the corresponding model selection criteria across all candidate models.

**Table 5:** Number of selected times as the best-model and the second-best model based on DIC, LPML, WAIC, and LOO in Simulation 3.

	**Times of selected as the best model**					**Times of selected as the second best model**				
	**1PGlogit**	**Rasch**	**1PNO**	**CLLM**	**3PL**	**1PGlogit**	**Rasch**	**1PNO**	**CLLM**	**3PL**
**True model: 3PL model**										
DIC LPML WAIC LOO	12	0	0	0	38	38	0	0	0	12
	0	0	0	0	50	50	0	0	0	0
	0	0	0	0	50	50	0	0	0	0
	0	0	0	0	50	49	1	0	0	0
	**1PGlogit**	**Rasch**	**1PNO**	**CLLM**	**3PL**	**1PGlogit**	**Rasch**	**1PNO**	**CLLM**	**4PL**
**True model: 4PL model**										
DIC LPML WAIC LOO	25	0	0	0	25	25		0	0	25
	0	0	0	0	50	50	0	0	0	0
	0	0	0	0	50	50	0	0	0	0
	0	0	0	0	50	50	0	0	0	0

Secondly, when the true model is the 4PL model, the results are nearly identical to those under the 3PL model. The 4PL model performs the best based on LPML, WAIC, and LOO, and is selected as the optimal model in all 50 repetitions. The second-best model is consistently the 1PGlogit model. In terms of DIC value, the average for the 4PL model is the lowest, but in 25 out of 50 repetitions, the 1PGlogit model is chosen as the best. As illustrated by the boxplot in Figure 7, the model selection criteria of the 1PGlogit model are significantly lower than those of the other one-parameter models. Figure 8 displays the ICCs of the first item. Aside from the 4PL model, the ICC of the 1PGlogit model demonstrates the best fitting performance, suggesting that this flexible 1PGlogit model provides a well-fitted representation of the guessing behavior and slipping behavior assumed in the 4PL model, which affects the lower and upper asymptotes.

In summary, the 1PGlogit model demonstrates superior fitting performance for asymmetric models compared to other one-parameter models. This model enhances flexibility by adjusting the parameter **λ** to fit the upper and lower asymptotes. However, we observed that DIC sometimes failed to identify the true model in this simulation, as was also the case when Rasch was the true model in Simulation 2. According to Luo and Al-Harbi (2016), within the dichotomous IRT framework, the performances of WAIC and LOO surpass that of DIC. Therefore, in light of the findings of this paper, we recommend giving greater consideration to LPML, WAIC, and LOO criteria when selecting models.

## 5. Real data

For this example, we use the 2015 computer-based PISA science data. Out of all the countries that took part in the computer-based science assessment, we selected data from the United States of America (USA). The initial sample consisted of 685 students, but 76 students were excluded due to Not Reached (original code 6) or No Response (original code 9) outcomes. These Not Reached and No Response results were treated as missing data. Therefore, the final sample size stands at 609 students, for whom the response data is available. The 11 items were scored on a dichotomous scale. We utilize six different models to fit the PISA dataset. This includes two symmetric models, namely the Rasch and the 1PNO models, in conjunction with three asymmetric models: the CLLM, the 3PL model, the 4PL model, and our generalized logistic model, known as the 1PGlogit model. During the process of estimation, we employ the same prior probabilities for the unknown parameters as used in simulations 2 and 3. Throughout all Bayesian computations, we generate 5,000 MCMC samples after a burn-in period of 4,000 iterations for each model to compute all the posterior estimates. The convergence of the chains is assured by evaluating the PSRF values ($\hat{R}$). For each model, the PSRF values of all parameters, both item and person, are observed to be under 1.1.

**Figure 9:**
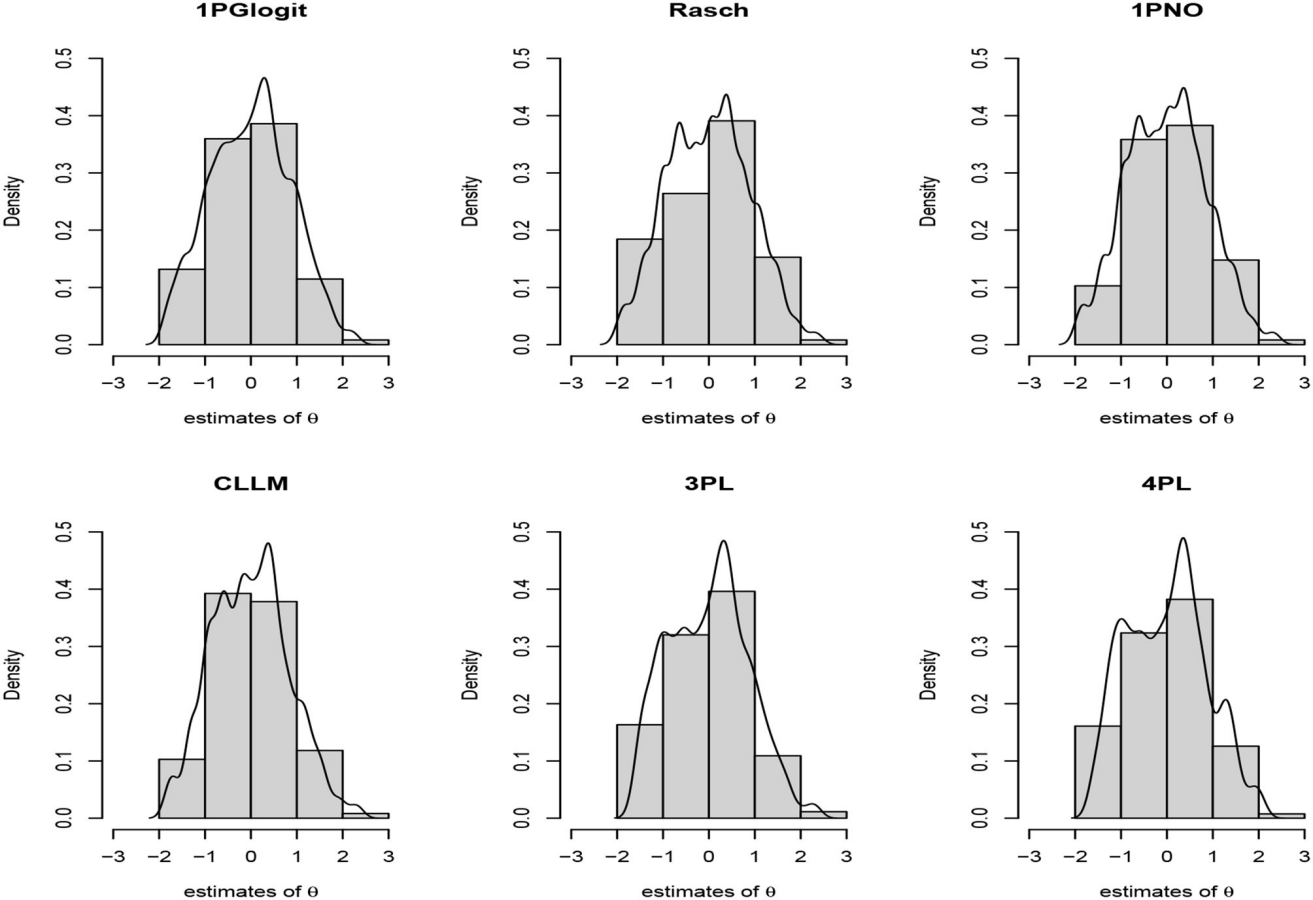
Item characteristic curve (ICC) of all items based on 1PGlogit model for the real data.

**Figure 10:**
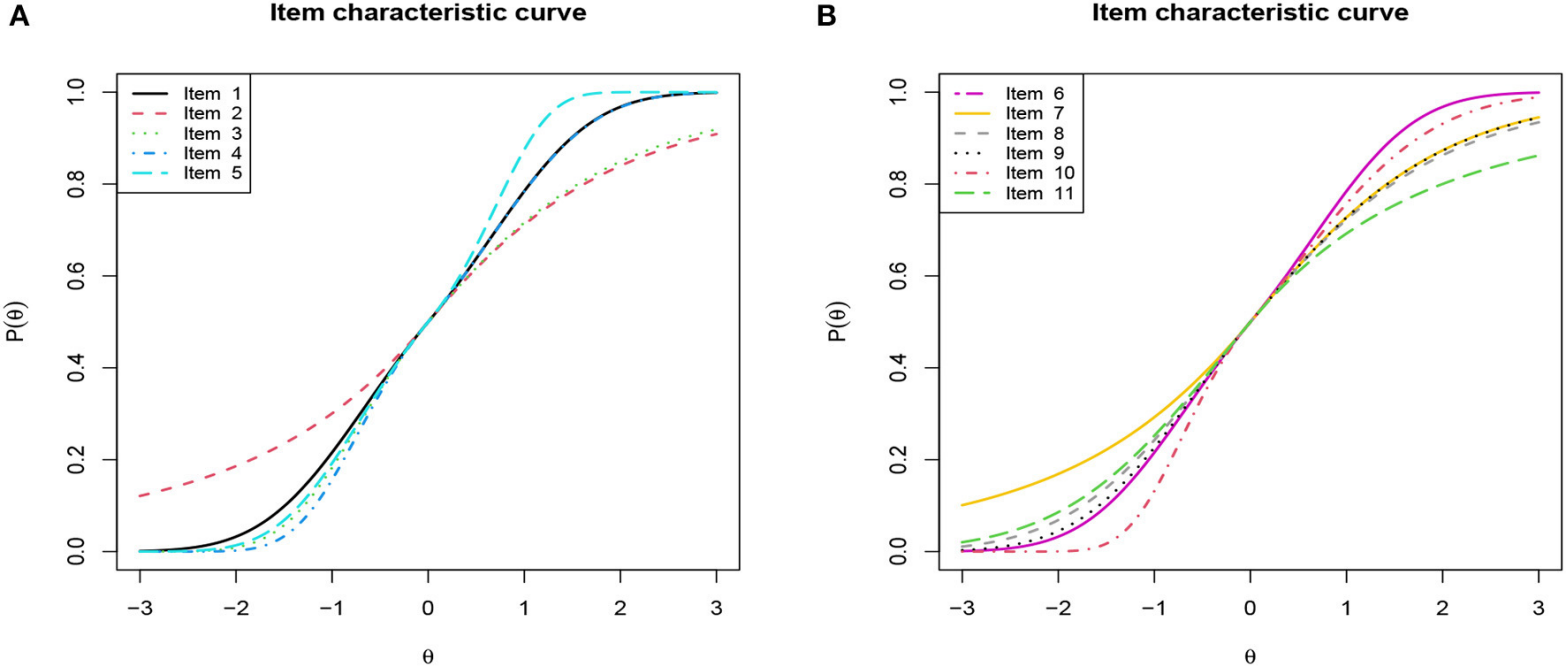
Item characteristic curve (ICC) of all items based on 1PGlogit model for the real data. **(A)** ICC of Item 1–5. **(B)** ICC of Item 6–11.

First, we depicted the frequency distribution histogram of the estimated ability parameter **θ** values across different models in Figure 9, and fitted their respective distribution curves. From this, it is apparent that the distributions of the estimated ability parameters remain largely consistent across the varied models. Upon examining the fitted distributions of the estimated **θ**, it can be observed that the ability distribution under the 1PGlogit model is closest to a normal distribution. The **θ** distributions under the Rasch and 1PNO models are notably similar, while the **θ** distributions under the 3PL model are more analogous to those of the 4PL model. Next, we provide detailed results of the Bayesian model assessment for the PISA dataset in Table 6. All these criteria indicate that the 1PGlogit model fits the data best among the six models. The second-best fitting model tends to be either the 3PL model or the 4PL model, both of which demonstrate similar fitting effects, while the three one-parameter IRT models show a notably inferior fit compared to the others. Hence, we surmise that the data shows a preference for flexible asymmetrical models. Based on the results of the model assessment, we will proceed with the best fitting 1PGlogit model for the analysis of the PISA data. In Table 7, we provide the estimated values of parameters in the 1PGlogit model, including the SD, 95% highest posterior density interval (HPDI), and $\hat{R}$ for each parameter. It is evident from the $\hat{R}$ values that the Markov chain has achieved convergence. Examining the estimated parameter values, we note firstly that item 6 is the most difficult, with β_6_ = 2.4805, while item 11 is the easiest, with β_11_ = −2.5073. Moreover, for the parameter **λ_1_**, the values are mostly small, except for item 5 which exceeds 1, suggesting that the tail of this item's ICC approaches the upper asymptote more quickly. Conversely, the estimated values for **λ_2_** are generally larger and positive, such as for item 10, which exceeds 1, indicating a rapid approach to the lower asymptote for the tail of its ICC. Lastly, we have plotted the ICCs for all the items in Figure 10. From Figure 10, it can be seen that for item 2, there appears to be some guessing behavior among low ability students, as they have a certain probability of answering the item correctly even with very low ability. Conversely, high ability students may exhibit slipping behavior, as even with relatively high ability, their probability of answering correctly is only around 90%. In contrast, for item 5, students with ability values below 2 have virtually no chance of answering correctly, while those with ability values exceeding 1.5 have almost no chance of answering incorrectly. In essence, the 1PGlogit model can deliver robust data fitting and outstanding interpretability.

**Table 6:** Values of DIC, LPML, WAIC, and LOO for 1PGlogit, Rasch, 1PNO, CLLM, 3PL, and 4PL models for the real data.

**Model**	**DIC**	**LPML**	**WAIC**	**LOO**
1PGlogit	**6464.903**	**–3257.137**	**6454.856**	**6499.141**
Rasch	6689.912	–3341.841	6679.935	6684.846
1PNO	6647.231	–3330.087	6651.59	6661.054
CLLM	6708.316	–3387.79	6742.526	6771.13
3PL	6528.033	–3276.638	6528.809	6552.125
4PL	6552.333	–3275.981	6528.991	6550.834

The bold values represent the minimum values of the corresponding model selection criteria across all candidate models.

**Table 7:** Parameter estimates for all items based on the 1PGlogit model in real data.

	**Estimate**	**SD**	**HPDI**	** $\hat{R}$ **
**β**				
Item 1	–0.3259	0.1178	[–0.5528, –0.1009]	1.0027
Item 2	0.7981	0.1544	[0.5035, 1.1023]	1.0001
Item 3	0.6522	0.1241	[0.4250, 0.9101]	1.0024
Item 4	–0.1680	0.1069	[-0.3772, 0.0352]	1.0006
Item 5	–0.7112	0.1055	[–0.9126, –0.4937]	1.0015
Item 6	2.4805	0.2461	[2.0107, 2.9589]	0.9996
Item 7	0.0470	0.1325	[–0.2003, 0.3080]	1.0000
Item 8	–0.5728	0.1416	[–0.8627, –0.3006]	0.9999
Item 9	0.9687	0.1407	[0.7115, 1.2589]	1.0010
Item 10	1.5533	0.1419	[1.2907, 1.8341]	1.0010
Item 11	–2.5073	0.3414	[–3.2047, –1.8844]	1.0001
**λ_1_**				
Item 1	0.4927	0.2843	[–0.0540, 1.0522]	1.0018
Item 2	–0.2222	0.4696	[–0.9962, 0.6246]	1.0018
Item 3	–0.1674	0.4650	[–0.9995, 0.6549]	1.0023
Item 4	0.4861	0.3014	[–0.0436, 1.1152]	1.0006
Item 5	1.2090	0.3863	[0.5059, 1.9562]	1.0010
Item 6	0.1382	0.6449	[–0.9873, 1.3015]	1.0008
Item 7	–0.0362	0.3830	[–0.8312, 0.7113]	1.0020
Item 8	–0.0904	0.2658	[–0.6508, 0.4159]	1.0014
Item 9	–0.0398	0.5547	[–0.9744, 0.9865]	1.0017
Item 10	0.2537	0.6769	[–0.9908, 1.4677]	1.0000
Item 11	–0.4990	0.1685	[–0.8577, –0.1966]	1.0016
**λ_2_**				
Item 1	0.4891	0.5216	[–0.5493, 1.5489]	1.0029
Item 2	–0.3930	0.2545	[–0.9086, 0.0654]	1.0020
Item 3	0.9644	0.5037	[0.0353, 1.9567]	1.0046
Item 4	0.7601	0.3364	[0.1513, 1.4065]	1.0017
Item 5	0.6816	0.6276	[–0.4691, 1.9689]	1.0003
Item 6	0.7804	0.3510	[0.2051, 1.4938]	0.9994
Item 7	–0.2759	0.3416	[–0.9636, 0.3079]	0.9997
Item 8	0.2539	0.5746	[–0.9122, 1.2491]	1.0002
Item 9	0.3990	0.2278	[–0.0012, 0.8625]	1.0019
Item 10	1.1567	0.0094	[0.4972, 1.9112]	1.0010
Item 11	0.1632	0.6592	[–0.9988, 1.3891]	1.0000

## 6. Discussion

This paper discusses a generalized one-parameter IRT model, the 1PGlogit model, which can encompass commonly-used IRT models such as the Rasch, 1PNO, and the recently proposed CLLM as its submodels. Owing to its adjustable parameter **λ**, it exhibits high flexibility, which enables control over the rate at which it approaches the upper and lower asymptotes of the ICC. In this paper, we first examine the accuracy of the model in parameter recovery using the Stan program. Subsequently, we investigate its performance in fitting data generated by other one-parameter IRT models. Finally, we delve deeper into its effectiveness in fitting asymmetric 3PL and 4PL models.

From the simulation results, we can draw the following conclusions. Firstly, the estimates generated by Stan are consistent with the large sample properties and exhibit excellent parameter recovery accuracy. The difficulty parameter demonstrates the highest estimation precision, followed by **λ** and θ. Secondly, the 1PGlogit model showcases commendable fitting performance for data generated by its various submodels. It ranks as the best model in terms of fitting performance, with the exception of the true model. Finally, the 1PGlogit model presents an outstanding fit for data generated by the asymmetric 3PL and 4PL models, markedly superior to other one-parameter IRT models. The 1PGlogit model can more accurately recover the shape of the ICC of the 3PL/4PL model.

In summary, the 1PGlogit model is a highly flexible and generalized model that encompasses Rasch, 1PNO, and CLLM as its submodels. Its parameter **λ** adjusts the speed at which the ICC curve approaches the upper and lower asymptotes. A larger λ_1_ results in a quicker approach to the upper asymptote, and a larger λ_2_ results in a swifter approach to the lower asymptote. As such, the 1PGlogit model can effectively accommodate the assumptions of guessing and slipping behavior in the 3PL and 4PL models, which would otherwise cause the upper and lower asymptotes to diverge from 1 and 0, respectively. However, the 1PGlogit model also has its limitations. Firstly, the constraint that its parameter **λ** must be greater than -1 may inhibit the model's ability to depict behaviors on the ICC where the asymptotes significantly diverge from 1 and 0. Secondly, although the 1PGlogit model is a generalized model that includes other one-parameter IRT models, the introduction of the new parameter **λ** adds complexity to the model, and the estimation accuracy of 1PGlogit is slightly lower than that of other one-parameter models. Moreover, the introduction of **λ** may also introduce some identifiability issues to the model, where **λ** and θ might mutually influence each other.

In conclusion, we would like to propose some directions for future work. The 1PGlogit model is a flexible and generalized model, and this paper merely provides an initial exploration of its advantages in fitting various types of data. We believe there is significant potential for its further development and application, such as extending the 1PGlogit model to higher-order IRT models, graded response models, multilevel IRT models, and longitudinal IRT models, among others. Therefore, in our future research, we will dedicate ourselves to the advancement and application of the 1PGlogit model in these proposed areas. Moreover, a wealth of scholarly work has been dedicated to formulating link functions for binary and ordinal response data. Notable contributions in this field have been made by Aranda-Ordaz (1981), Guerrero and Johnson (1982), Stukel (1988), Kim et al. (2008), Wang and Dey (2010), and Jiang et al. (2014), among others. It is worth exploring whether these existing link functions can be directly applied to the field of IRT. We intend to investigate this possibility in our future work.

## Data availability statement

Publicly available datasets were analyzed in this study. This data can be found at: https://www.oecd.org/pisa/data/.

## Author contributions

XW and JZ completed the writing of this article. XW, JZ, and JL completed the article revisions. JL provided original thoughts. JZ, JL, GC, and NS provided key technical support. All authors contributed to the article and approved the submitted version.

## References

[B1] Aranda-Ordaz F. J. (1981). On two families of transformations to additivity for binary response data. Biometrika 68, 357–363. 10.1093/biomet/68.2.357

[B2] Arenson E. A. Karabatsos G. (2018). A Bayesian beta-mixture model for nonparametric IRT (BBM-IRT). J. Modern Appl. Stat. Methods 17, 1–18. 10.22237/jmasm/1531318047

[B3] Baker F. B. Kim S. H. (2004). Item Response Theory: Parameter Estimation Techniques, 2nd Edn. CRC Press. 10.1201/9781482276725

[B4] Bechtel G. G. (1985). Generalizing the Rasch model for consumer rating scales. Market. Sci. 4, 62–73. 10.1287/mksc.4.1.6231274238

[B5] Bezruczko N. (2005). Rasch Measurement in Health Sciences. Maple Grove, MN: Jam Press.

[B6] Bolfarine H. Bazan J. L. (2010). Bayesian estimation of the logistic positive exponent IRT model. J. Educ. Behav. Stat. 35, 693–713. 10.3102/1076998610375834

[B7] Brooks S. P. Gelman A. (1998). Alternative methods for monitoring convergence of iterative simulations. J. Comput. Graph. Stat. 7, 434–455. 10.1080/10618600.1998.10474787

[B8] Chen M. H. Dey D. K. Shao Q. M. (1999). A new skewed link model for dichotomous quantal response data. J. Am. Stat. Assoc. 94, 1172–1186. 10.1080/01621459.1999.10473872

[B9] Chen M. H. Dey D. K. Wu Y. (2002). On robustness of choice of links in binomial regression. Calcutta Stat. Assoc. Bull. 53, 145–164. 10.1177/0008068320020113

[B10] Duncan K. MacEachern S. (2008). Nonparametric Bayesian modelling for item response. Stat. Modell. 8, 41–66. 10.1177/1471082X0700800104

[B11] Embretson S. E. Reise S. P. (2000). Item Response Theory for Psychologists. Lawrence Erlbaum Associates.

[B12] Ferguson G. A. (1942). Item selection by the constant process. Psychometrika 7, 19–29. 10.1007/BF02288601

[B13] Geisser S. Eddy W. F. (1979). A predictive approach to model selection. J. Am. Statist. Assoc. 74, 153–160. 10.1080/01621459.1979.10481632

[B14] Geman S. Geman D. (1984). Stochastic relaxation, Gibbs distributions, and the Bayesian restoration. IEEE Trans. Pattern Anal. Mach. Intell. 6, 721–741. 22499653 10.1109/tpami.1984.4767596

[B15] Goldstein H. (1980). Dimensionality and the fitting of unidimensional item response models to multidimensional data. Appl. Psychol. Meas. 4, 355–365. 20954056

[B16] Guerrero V. M. Johnson R. A. (1982). Use of the Box-Cox transformation with binary response models. Biometrika 69, 309–314. 10.1093/biomet/69.2.309

[B17] Hoffman M. D. Gelman A. (2014). The No-U-Turn sampler: adaptively setting path lengths in Hamiltonian Monte Carlo. J. Mach. Learn. Res. 15, 1593–1623. Available online at: https://jmlr.org/papers/v15/hoffman14a.html

[B18] Ibrahim J. G. Chen M. H. Sinha D. (2001). Bayesian Survival Analysis. New York, NY: Springer.

[B19] Jiang X. Dey D. K. Prunier R. Wilson A. M. Holsinger K. E. (2014). A new class of flexible link functions with application to species co-occurrence in Cape floristic region. Ann. Appl. Stat. 7, 2180–2204 10.1214/13-AOAS663

[B20] Karabatsos G. (2016). Bayesian nonparametric IRT, in Handbook of Item Response Theory, Vol. 1, ed van der Linden W. J. (Boca Raton, FL: Chapman and Hall/CRC), 323–336.

[B21] Kim S. Chen M.-H. Dey D. K. (2008). Flexible generalized t-link models for binary response data. Biometrika 95, 93–106. 10.1093/biomet/asm079

[B22] Lawley D. N. (1943). On problems connected with item selection and test construction. Proc. R. Soc. Edinburgh 61, 273–287. 10.1017/S008045410000628236420000

[B23] Lawley D. N. (1944). The factorial invariance of multiple item tests. Proc. R. Soc. Edinburgh, 62-A, 74–82. 10.1017/S0080454100006440

[B24] Lord F. M. (1952). A theory of test scores. Psychometr. Monogr. 7, 1–100.

[B25] Lord F. M. (1953). An application of confidence intervals and of maximum likelihood to the estimation of an examinee's ability. Psychometrika 18, 57–75. 10.1007/BF02289028

[B26] Lord F. M. (1980). Applications of Item Response Theory to Practical Testing Problems. Hillsdale, NJ: Lawrence Erlbaum Associates.

[B27] Lord F. M. Novick M. R. (1968). Statistical Theories of Mental Test Scores. Menlo Park, CA: Addison-Wesley.

[B28] Lucke J. F. (2014). Positive trait item response models, in New Developments in Quantitative Psychology: Presentations from the 77th Annual Psychometric Society Meeting, eds Millsap R. E. van der Ark L. A. Bolt D. M. Woods C. M. (New York, NY: Routledge), 199–213. 10.1007/978-1-4614-9348-8_13

[B29] Lunn D. J. Thomas A. Best N. Spiegelhalter D. (2000). WinBUGS-a Bayesian modelling framework: concepts, structure, and extensibility. Stat. Comput. 10, 325–337. 10.1023/A:1008929526011

[B30] Luo Y. Al-Harbi K. (2016). Performances of LOO and WAIC as IRT model selection methods, in Paper presented at the International Meeting of Psychometric Society (Ashville, NC).

[B31] Luo Y. Jiao H. (2018). Using the Stan program for Bayesian item response theory. Educ. Psychol. Meas. 78, 384–408. 10.1177/0013164417693666 30140099 PMC6096466

[B32] Luzardo M. Rodriguez P. (2015). A nonparametric estimator of a monotone item characteristic curve, in Quantitative Psychology Research, eds van der Ark L. A. Bolt D. Wang W. C. Douglas A. Chow S. M. (Cham: Springer International Publishing), 99–108. 10.1007/978-3-319-19977-1_8

[B33] Magnus B. E. Liu Y. (2018). A zero-inflated Box-Cox normal unipolar item response model for measuring constructs of psychopathology. Appl. Psychol. Meas. 42, 571–589. 10.1177/0146621618758291 30237647 PMC6140303

[B34] Metropolis N. Rosenbluth A. W. Rosenbluth M. N. Teller A. H. Teller E. (1953). Equation of state calculations by fast computing machines. J. Chem. Phys. 21, 1087–1092. 10.1063/1.1699114 41342507

[B35] Moral F. J. Rebollo F. J. (2017). Characterization of soil fertility using the Rasch model. J. Soil Sci. Plant Nutr. 10.4067/S0718-9516201700500003527315006

[B36] Mosier C. L. (1940). Psychophysics and mental test theory: fundamental postulates and elementary theorems. Psychol. Rev. 47, 355–366. 10.1037/h0059934

[B37] Mosier C. L. (1941). Psychophysics and mental test theory. II. The constant process. Psychol. Rev. 48, 235–249. 10.1037/h0055909

[B38] Neal R. M. (2011). MCMC using Hamiltonian dynamics, in Handbook of Markov Chain Monte Carlo, Vol. 2, ed Brooks S. (Boca Raton, FL: CRC Press/Taylorand Francis), 113–162. 10.1201/b10905-6

[B39] Plummer M. (2003). JAGS: a program for analysis of Bayesian graphical models using Gibbs sampling, in Proceedings of the 3rd International Workshop on Distributed Statistical Computing (DSC 2003) (Vienna), 20–22.

[B40] Qin L. (1998). Nonparametric Bayesian models for item response data (Unpublished doctoral dissertation). The Ohio State University, Columbus, OH, United States.

[B41] R Core Team (2019). R: A Language and Environment for Statistical Computing. R Foundation for Statistical Computing, Vienna.

[B42] Rasch G. (1960). Probabilistic Model for Some Intelligence and Achievement Tests. Copenhagen: Danish Institute for Educational Research.

[B43] Richardson M. W. (1936). The relation between the difficulty and the differential validity of a test. Psychometrika 1, 33–49. 10.1007/BF02288003

[B44] Samejima F. (1997). Ability estimates that order individuals with consistent philosophies, in Paper presented at the 1997 Meeting of the American Educational Research Association (Chicago, IL).

[B45] Samejima F. (1999). Usefulness of the logistic positive exponent family of models in educational measurement, in Paper presented at the 1999 Meeting of the American Educational Research Association (Montreal, QC).

[B46] Samejima F. (2000). Logistic positive exponent family of models: virtue of asymmetric item characteristics curves. Psychometrika 65, 319–335. 10.1007/BF02296149

[B47] Shim H. Bonifay W. Wiedermann W. (2022). Parsimonious asymmetric item response theory modeling with the complementary log-log link. Behav. Res. Methods 55, 200–219. 10.3758/s13428-022-01824-5 35355241

[B48] Spiegelhalter D. J. Best N. G. Carlin B. P. Van Der Linde A. (2002). Bayesian measures of model complexity and fit. J. R. Stat. Soc. Ser. B Stat. Methodol. 64, 583–639. 10.1111/1467-9868.00353

[B49] Spiegelhalter D. J. Thomas A. Best N. G. Lunn D. (2010). OpenBUGS Version 3.1.1 User Manual.

[B50] Stan Development Team (2019). Stan Modeling Language: User's Guide and Reference Manual.

[B51] Stukel T. A. (1988). Generalized logistic models. J. Am. Stat. Assoc. 83, 426–431. 10.1080/01621459.1988.10478613

[B52] Tucker L. R. (1946). Maximum validity of a test with equivalent items. Psychometrika 11, 1–13. 10.1007/BF02288894 21018318

[B53] van der Linden W. J. Hambleton R. K. (1997). Handbook of Modern Item Response Theory. New York, NY: Springer. 10.1007/978-1-4757-2691-6

[B54] Vehtari A. Gelman A. Gabry J. (2017). Practical Bayesian model evaluation using leave-one-out cross-validation and WAIC. Stat. Comput. 27, 1413–1432. 10.1007/s11222-016-9696-4

[B55] Wang X. Dey D. K. (2010). Generalized extreme value regression for binary response data: An application to B2B electronic payments system adoption. Ann. Appl. Stat. 4, 2000–2023. 10.1214/10-AOAS354

[B56] Watanabe S. Opper M. (2010). Asymptotic equivalence of bayes cross validation and widely applicable information criterion in singular learning theory. J. Mach. Learn. Res. 11, 3571–3594. Available online at: https://www.jmlr.org/papers/volume11/watanabe10a/watanabe10a.pdf

[B57] Wright B. D. (1977). Solving measurement problems with the Rasch mode. J. Educ. Measure. 14, 97–116. 10.1111/j.1745-3984.1977.tb00031.x30863344

[B58] Zhang J. Zhang Y. Y. Tao J. Chen M. H. (2022). Bayesian item response theory models with flexible generalized logit links. Appl. Psychol. Meas. 46, 382–405. 10.1177/01466216221089343 35812812 PMC9265488

