# Supplementary material for: Exploration and analysis of a generalized one-parameter item response model with flexible link functions
Source: Front Psychol. 2023 Aug 30;14:1248454. doi: 10.3389/fpsyg.2023.1248454 (PMC10498775; doi:10.3389/fpsyg.2023.1248454)
Supplement: Supplementary file 1 [file Data_Sheet_1.PDF]

Online Supplementary Materials: Further Explanation and Elaboration on This Study

Manuscript ID: 1248454

1) Discussions on Potential Scale Reduction Factor in Multiple Repeated Experiments

Indeed, in our simulation studies, the Potential Scale Reduction Factor (PSRF; also known as $\widehat{R}$) we are interested in is not an average of $\widehat{R}$ s from 50 repeated experiments for the parameter of interest. Instead, we obtain a $\widehat{R}$ for the parameter of interest from each of the 50 repeated experiments. As a result, from 50 repetitions, we obtain 50 $\widehat{R}$ s. To visualize the multitude of PSRFs from these 50 repetitions, we have employed a boxplot in Figure 3 of our original manuscript.

Taking difficulty parameter $\boldsymbol{\beta}$ as an example, under the scenario where N is 1000 and J is 20, we have 20 $\boldsymbol{\beta}$ s for the 20 items. Each $\boldsymbol{\beta}$ generates 50 $\widehat{R}$ values from the 50 repeated simulations. Consequently, in Figure 3, the boxplot for the $\boldsymbol{\beta}$ parameter encapsulates a total of 20×50, which is 1000 $\widehat{R}$ values. Similarly, for the $\boldsymbol{\theta}$ parameter, it encompasses 1000 (the ability parameters for 1000 examinees) times 50 (from 50 repeated simulations), resulting in 50000 $\widehat{R}$ values.

2) Discussion on the Range of Shape Parameter (λ) Values

We will elaborate on why $\boldsymbol{\lambda}$ needs to be greater than -1 using the Stukel's link as an example, and providing a detailed explanation through the proof of Theorem 1. Similarly, in our study, the Glogit IRT link and the Stukel's link are identical, hence the methods and processes of proof are the same. Next, we present the following cumulative distribution function (CDF) employed for Stukel's model.

$$F_{sk}(\eta)=\begin{cases}\left[1+\exp\left(\frac{\exp(\lambda_1\eta)-1}{\lambda_1}\right)\right]^{-1} & \text{if } \lambda_1>0 \text{ and } \eta\geq 0\\ \left[1+\exp\left(\frac{\log(1-\lambda_1\eta)}{\lambda_1}\right)\right]^{-1} & \text{if } \lambda_1<0 \text{ and } \eta\geq 0\\ \left[1+\exp\left(\frac{\exp(-\lambda_2\eta)-1}{\lambda_2}\right)\right]^{-1} & \text{if } \lambda_2>0 \text{ and } \eta\leq 0\\ \left[1+\exp\left(-\frac{\log(1+\lambda_2\eta)}{\lambda_2}\right)\right]^{-1} & \text{if } \lambda_2<0 \text{ and } \eta\leq 0\end{cases} \quad (1)$$

Theorem 1 Let $W\sim F_{sk}$, Then, the r th moment of $W(r>0)$ exists, i.e.,

$$E\left[|W|^r\right]<\infty,\ \text{if } \lambda_1>-\frac{1}{r} \text{ and } \lambda_2>-\frac{1}{r}.$$

Proof: It is readily apparent that the form of the probability density function is inherent to Stukel's model

$$f_{sk}(\eta)=\frac{dF_{sk}(\eta)}{d\eta}=\frac{\exp\left(h_\lambda(\eta)\right)}{\left[1+\exp\left(h_\lambda(\eta)\right)\right]^2}\frac{dh_\lambda(\eta)}{d\eta}.$$

Thus, the r th moment of W is given by

$$\begin{aligned}E\left(|W|^r\right)&=\int_{-\infty}^{\infty}|\eta|^r f_{sk}(\eta)d\eta\\ &\overset{t=h_\lambda}{=}\int_{-\infty}^{\infty}\left|h_\lambda^{-1}(t)\right|^r\frac{e^t}{(1+e^t)^2}dt\\ &=\int_0^{\infty}\left|h_\lambda^{-1}(t)\right|^r\frac{e^t}{(1+e^t)^2}dt+\int_{-\infty}^{0}\left|h_\lambda^{-1}(\eta)\right|^r\frac{e^t}{(1+e^t)^2}dt\\ &\overset{\text{def}}{=} I_1+I_2,\end{aligned}$$

where h_λ^{-1} is the inverse function of h_λ. Next, we consider two cases for I_1.

Case (i) $\lambda_1>0$: Using (1), we have

$$h_\lambda^{-1}(t)=\frac{\log\left(\lambda_1 t+1\right)}{\lambda_1}.$$

Thus, it is easy to verify that

$$I_1=\int_0^{\infty}\left|\frac{\log\left(\lambda_1 t+1\right)}{\lambda_1}\right|^r\frac{e^t}{(1+e^t)^2}dt<\infty.$$

Case (ii) $\lambda_1<0$: Similar to Case (i), the inverse function of h_λ can be written as

$$h_\lambda^{-1}(t)=\frac{1-\exp\left(-\lambda_1 t\right)}{\lambda_1},$$

and

$$I_1 = \int_0^{\infty} \left| \frac{1-\exp(-\lambda_1 t)}{\lambda_1} \right|^r \frac{e^t}{(1+e^t)^2} dt$$
$$= \int_0^{\infty} \left| \frac{1-\exp(\lambda_1 t)}{\lambda_1} \right|^r \frac{\exp[-(1+r\lambda_1)t]}{(1+\exp(-t))^2} dt < \infty$$

If $1+r\lambda_1 > 0$. Note that $1+r\lambda_1 > 0$ implies $\lambda_1 > -\frac{1}{r}$. Combining **(i)** and **(ii)** leads to that $I_1 < \infty$ if $\lambda_1 > -\frac{1}{r}$. Similarly, we can show that $I_2 < \infty$ if $\lambda_2 > -\frac{1}{r}$. This completes the proof. We note that the conditions given in Theorem 1 are not only sufficient but also necessary. From Theorem 1, the first moment of W exists if $\lambda_1 > -1$ and $\lambda_2 > -1$, and the second moment of W exists if $\lambda_1 > -\frac{1}{2}$ and $\lambda_2 > -\frac{1}{2}$. Thus, $Var(W)$ is finite if and only if $\lambda_1 > -\frac{1}{2}$ and $\lambda_2 > -\frac{1}{2}$. Based on the discussions centered around the Stukel's link, in this study, we impose the restrictions $\lambda_1 > -1$ and $\lambda_2 > -1$ to guarantee the existence of the first moment. For detailed proofs, please refer to reference Chen, Dey, and Wu (2002).

3) **Compared to the Traditional Generalized Logistic Model, What is the Significance and Value of the Glogit IRT Model Proposed in This Research?**

We will elucidate the value and practical significance of our Glogit IRT link model from six aspects. While both Stukel regression with linear prediction η and the 1PGlogit model with linear prediction $\beta-\theta$ share a common link function foundation, they fundamentally differ in their applications and interpretations, particularly from the perspective of educational psychology.

Purpose and Scope of Application

Stukel regression (1988) is a versatile statistical method that applies to many fields. In educational psychology, it's often used to examine the impact of different factors (various predictors) on dichotomous outcomes. It can handle complex relationships by including multiple predictors and interaction terms. For

instance, you might use Stukel regression to investigate whether factors like parental education level, hours spent on homework, or school quality influence the likelihood of a student passing an exam (a binary outcome: pass/fail).

On the contrary, the 1PGlogit model, by design, is more specific to the realm of psychometrics, primarily utilized for the analysis and construction of educational tests and assessments. The goal of a 1PGlogit model is to determine the properties of individual test items, specifically, their difficulty and discriminatory power. This information can help in creating tests that accurately measure the latent abilities of a student body with a variety of skill levels.

Parameters

The Stukel regression model provides regression coefficients for each predictor variable. These coefficients are typically interpreted as the change in the log-odds of the outcome for a one-unit change in the predictor. This allows you to quantify and compare the impacts of different factors. For example, you might find that an extra hour of homework per week has twice the impact on the likelihood of passing an exam compared to attending a higher-quality school. However, the 1PGlogit model is constructed is constructed using the latent ability parameter and the difficulty parameter. The difficulty parameter is used to describe the relative difficulty of individual items in a test or assessment. In a nutshell, it provides an estimate of the point along the latent trait or ability continuum at which a respondent has a 50% chance of answering an item correctly. A higher difficulty parameter means the item is more difficult (requires higher ability to have a 50% chance of correct response), while a lower difficulty parameter means the item is easier (requires lower ability to have a 50% chance of correct response).

Item Characterization

Stukel regression: Does not provide information about individual items in a test.

1PGlogit model: Provides valuable information about the difficulty of each item. This is crucial in educational and psychological testing for understanding which items are more difficult between ability levels, and how to improve the test.

Assumptions

Stukel regression: There are no assumptions required when utilizing Stukel regression.

1PGlogit model: In applying the classic 1PGlogit model within the framework of IRT, three fundamental assumptions need to be satisfied: 1) Responses across individuals are independent of each other; 2) Items are independent of each other, with no relationships between them; 3) The relationship between individuals and items is independent, meaning there is no mutual influence when an individual responds to different items.

Test Adaptivity:

Stukel regression: Not well suited for adaptive testing because it doesn't account for item-specific parameters.

1PGlogit model: The output can be used to create adaptive tests, where the difficulty of the next item an individual sees is based on their previous responses. This makes testing more efficient and precise, especially useful in large-scale assessments.

Scale Invariance:

Stukel regression: Predictions depend on the specific predictors used in the model. This means that changing the test items or predictors would change the predicted outcomes.

1PGlogit model: Estimates of individuals' latent traits are invariant to the particular set of items used (provided the items fit the model well). This means that an individual's estimated ability would be the same regardless of which items from an item pool were used in the test.

In summary, although it may appear that Stukel regression and 1PGlogit model are simply transformations of η into $\beta-\theta$, there are substantial differences in terms of their meanings and values. This is the primary reason why we are introducing the Stukel link into educational psychology in this study.

4) Implementable Stan Code for 1PGlogit Model

```
functions{
real h_lambda(real lambda1, real lambda2, real eta){
  if (eta > 0){
       if (lambda1 > 0)
              return (exp(lambda1 * eta) - 1) / lambda1;
       else if (lambda1 == 0)
              return eta;
       else //alpha1 < 0
              return -log(1 - lambda1 * eta) / lambda1;
    }
    else{ //eta <= 0
         if (lambda2 > 0)
              return -(exp(-lambda2 * eta) - 1) / lambda2;
       else if (lambda2 == 0)
              return eta;
       else //alpha2 < 0
              return log(1 + lambda2 * eta) / lambda2;
    }
  }
}
data{
```

```
int<lower=0> n_student;
int<lower=0> n_item;
int<lower=0,upper=1> Y[n_student,n_item];
}
parameters{
  vector[n_student] theta;
  vector[n_item] beta;
  vector<lower=-1>[n_item] lambda1;
  vector<lower=-1>[n_item] lambda2;
  real<lower=0> sig_beta;
  real<lower=0> sig_lambda;
}

model{
  theta ~ normal(0,1);
  beta ~ normal(0,sig_beta);
  lambda1 ~ normal(0,sig_lambda);
  lambda2 ~ normal(0,sig_lambda);
  sig_beta ~ cauchy(0,5);
  sig_lambda ~ cauchy(0,5);
  for(i in 1:n_student){
    for (j in 1:n_item){
        Y[i,j] ~ bernoulli_logit( h_lambda(lambda1[j], lambda2[j], (theta[i] - beta[j])));
    }
  }
}
```

```
generated quantities{
    vector[n_item] log_lik_Y[n_student];


  for (i in 1: n_student){
       for (j in 1: n_item){
           log_lik_Y[i,j] = bernoulli_logit_lpmf(Y[i,j] | h_lambda(lambda1[j], lambda2[j], (theta[i] -
beta[j])));
           }
    }
}
```

References

Chen, M. H., Dey, D. K., & Wu, Y. (2002). On robustness of choice of links in binomial regression. *Calcutta Statistical Association Bulletin*, *53*, 145-164.

Stukel, T. A. (1988). Generalized logistic models. *Journal of the American Statistical Association*, *83*(402), 426–431.
